# An enhanced turbulent flow of water-based optimization for optimal power flow of power system integrated wind turbine and solar photovoltaic generators

**DOI:** 10.1038/s41598-023-41749-3

**Published:** 2023-09-05

**Authors:** Amir Zahedibialvaei, Pavel Trojovský, Maryam Hesari-Shermeh, Ivana Matoušová, Eva Trojovská, Štěpán Hubálovský

**Affiliations:** 1 Department of Electrical and Computer Engineering, Tarbiat Modares University, Tehran, Iran; 2 Department of Mathematics, Faculty of Science, University of Hradec Králové, Rokitanského 62, Hradec Králové, 500 03 Czech Republic; 3 Department of Applied Cybernetics, Faculty of Science, University of Hradec Králové, Hradec Králové, Czech Republic

**Keywords:** Energy science and technology, Engineering, Mathematics and computing

## Abstract

This paper uses enhanced turbulent flow in water-based optimization (TFWO), specifically ETFWO, to achieve optimal power flow (OPF) in electrical networks that use both solar photovoltaic (PV) units and wind turbines (WTs). ETFWO is an enhanced TFWO that alters the TFWO structure through the promotion of communication and collaboration. Individuals in the population now interact with each other more often, which makes it possible to search more accurately in the search area while ignoring local optimal solutions. Probabilistic models and real-time data on wind speed and solar irradiance are used to predict the power output of WT and PV producers. The OPF and solution methods are evaluated using the IEEE 30-bus network. By comparing ETFWO to analogical other optimization techniques applied to the same groups of constraints, control variables, and system data, we can gauge the algorithm’s robustness and efficiency in solving OPF. It is shown in this paper that the proposed ETFWO algorithm can provide suitable solutions to OPF problems in electrical networks with integrated PV units and WTs in terms of energy generation costs, improved voltage profiles, emissions, and losses, compared to the traditional TFWO and other proposed algorithms in recent studies.

## Introduction

### Motivation and incitement

The analytical approach used to determine the ideal topologies for the power system network is called optimal power flow (OPF). The primary goal of this problem is to minimize a specific objective function under the conditions of feasibility and security. OPF has been widely used in previous works due to its characteristics, including a highly constrained and large-scale nonlinear convex optimization test problem. Over the past few decades, many OPF formulations have been created to improve the performance of an electric power system that is subject to physical limitations^1^. Various names and multiple objective functions are used to describe the newly developed optimization problem. There are many different OPF solution methods, each with its math properties and processing needs^2^.

OPF optimization issues have been focused on recently due to the network’s quick deployment of distributed energy resources^2^. Traditional optimization techniques, such as Newton’s method, quadratic programming (QP) and nonlinear programming (NLP), show excellent convergence rates for solving OPF problems. However, these methods rely on theoretical assumptions unsuitable for real-world systems with non-smooth, non-differentiable, and non-convex cost functions. The above limitations can be overcome by using metaheuristics, which are based on a shared set of principles that allow the construction of solution algorithms. However, most metaheuristics take inspiration from nature, including stochastic components, and usually have many parameters that must be adapted to the situation.

### Literature review

We have to mention some papers in which a previously implemented artificial intelligence-based optimization method has been effectively used to solve OPF problems: Bouchekara et al.^3^ by an improved colliding bodies optimization (ICBO). Abd El-sattar et al.^4^ suggested an enhanced version of the salp swarm method (ISSA) to solve OPF, while Taher et al.^5^ proposed an enhanced version of the moth flame optimization (IMFO) technique to solve OPF. The behavior of moths, which always fly toward the moon, sparked the idea of moth flame optimization (MFO). The notion of MFO, in which moths' flight paths are altered to create new spirals around a flame, is the primary inspiration for IMFO. The IMFO algorithm's efficacy and reliability were verified using industry-standard IEEE 30bus, IEEE 57bus, and IEEE 118bus test systems^5^. The hybrid one proposed in^6^ uses the effective algorithms equilibrium optimizer (EO) and loss sensitivity factor (LSF). To improve the distribution system's performance as a whole, the weighted-sum multi-objective function (IMO) is designed to simultaneously reduce energy loss and voltage variation while increasing voltage stability. The standard IEEE 33-bus distribution system was used to test and validate the proposed method for optimal placement and sizing of WTGs and SMESs under conditions of time-varying voltage-dependent load models that included residential, industrial, commercial, and mixed loads, as well as variable wind speed. According to the simulations and numerical results, the distribution system's performance may significantly improve by integrating WTGs and SMES to reduce energy loss and voltage deviation while increasing voltage stability^6^. A variant of TLBO, called the teaching–learning-study-based optimizer (TLSO), was presented in^7^ to boost TLBO's worldwide optimization capabilities (TLSBO). The proposed improvement relied on incorporating a new technique into TLBO, dubbed the "studying strategy," in which each member leverages the knowledge of another randomly selected participant to solve the OPF issues^7^. Large-scale optimal reactive power dispatch (LS-ORPD) problems were the topic of^8^, which examined how Coulomb's and Franklin's laws (CFA) may be applied to find optimal solutions. The safe and reliable functioning of electrical power grids is being increasingly affected by ORPD issues, making this a vital area of research. Several restrictions from three IEEE standard power systems were used to verify the CFA optimizer's ability to resolve large-scale ORPD problems. When compared to existing solutions in the literature^8^, the proposed optimizer provides a more precise answer.

By testing on three IEEE systems with 30, 57, and 118 buses, Nguyen et al.^9^ used a social spider optimization (SSO) algorithm to independently optimize electricity generation fuel cost, power loss, polluted emission, voltage deviation, and L index in OPF; Duman al.^10^ used a symbiotic organism search (SOS) to the AC OPF problem and included test cases of stochastic wind, solar, and tidal energy systems.

The solutions were tested utilizing test systems for the IEEE 30bus and IEEE 118bus that used renewable energy sources, employing various locations depending on the chosen heat generating units, Kahraman et al.^2^ by an improved manta ray foraging optimizer (IMRFO) in which research was given on the creation of a technique that could identify the best set of solutions for a multi-objective OPF (MOOPF) whose objective functions were in conflict. For this reason, a strong and efficient technique was created using the Pareto archiving method based on crowding distance, Biswas et al.^11^ by an adaptive guided differential evolution (DE) (AGDE) combining stochastic solar and wind generation with traditional thermal power producers will solve OPF. Forecasting the output of solar and wind power was done using the Weibull and lognormal probability distribution functions, respectively, Ghasemi et al.^12^ by Lévy TLBO (LTLBO) that utilized the Lévy mutation approach for OPF problem control variables and was based on TLBO. On the conventional IEEE 30-bus and IEEE 57-bus test systems, the performance of this technique was examined and evaluated using several objective functions, Hazra et al.^13^ by a multi-objective particle swarm optimization (PSO) that to produce equally distributed Pareto optimal solutions while simultaneously minimizing the cost of generation and environmental damage, a diversity-preserving technique was used, Warid et al.^14^ by Jaya Algorithm (JAYA), which this algorithm did not require any algorithm-specific controlling parameters. In this study, the decrease in real power loss, enhancement of voltage stability, and minimization of generation costs were all considered when developing the OPF solution. Sarhan et al.^1^ used the turbulent flow of a water-based optimizer (TFWO) that, by considering 17 examples, were examined. The proposed algorithm was tried and investigated on the IEEE 30-bus and 57-bus systems, and goal functions like technical, economic, and emissions were taken into account. Herbadji et al.^15^ used the multi-objective ant lion algorithm (MOALA) to locate the multi-objective OPF's Pareto optimum front. The objective of this work was to simultaneously optimize four conflicting objective functions to arrive at good solutions to active and reactive OPF challenges, Narimani et al.^16^ by a hybrid PSO and shuffle frog leaping algorithm (SFLA) that the provided algorithm was used to 30, 57, and 118-bus test systems that had taken into account actual circumstances of power generation involving different constraints. Shi et al.^17^ by incorporating wind power, whereby a paradigm was put up to model the price of wind-generated power from a wind farm. The frequency distribution of wind farm power production that will serve as the foundation for estimating wind generation costs was established via Monte Carlo simulation, Elattar et al.^18^ by the modified moth swarm algorithm (MMSA), in which three objective functions—minimization of operational costs, minimization of transmission power loss, and enhancement of the voltage profile—were also taken into consideration for the OPF of a power system including stochastic wind power, Daryani et al.^19^ by the adaptive group search optimization (AGSO) that the various OPF factors were taken into account to create a precise multi-objective model. The first, second, and third-order objectives were the system total operation cost, total emissions, and $N-1$ security index, respectively, Khan et al.^20^ by grey wolf optimization (GWO), in which new contributions included the new objective functions and accompanying framework for optimizing generating cost while taking into account RES; in addition, the usage of GWO to tackle the non-convex OPF problem enhanced computing efficiency, Maheshwari et al.^21^ using the ant lion optimization (ALO) as well as the complete formulation of the OPF model taking into account WT and environmental emissions for optimum scheduling, planning, etc. Li et al.^22^ by a new improved adaptive DE, evaluate OPF on a modified IEEE 30-bus system, which integrates random sources of energy like solar and wind with traditional thermal power plants. El-Fergany^23^ applied the multi-objective version of GWO and DE algorithms to determine the ideal compromise point for multi-objective OPF (MOOPF) functions. The fuzzy-based Pareto front technique with the chaotic invasive weed was tested by, Ghasemi et al.^24^ on the standard IEEE 30-bus test system (optimizers CIWOs, based on chaos, were researched and assessed using various objective functions). Chen et al.^25^ showed, by the hybrid firefly-bat algorithm (HFBA), that ten MOOPF cases minimizing active power loss, total emission, and fuel cost are simulated on the IEEE 30-bus, IEEE 57-bus, and IEEE 118-bus systems to address the strictly limited MOOPF challenges. Islam et al.^26^ used the Harris hawks optimization (HHO) for resolving single-objective and multi-objective OPF problems to manage the emissions from thermal-producing sources. Weighted sums and the no-preference method have been used to resolve the various conflicting framed multi-objective functions. Avvari et al.^27^ used an evolutionary algorithm (EA) for OPF with four conflicting objectives, including minimization of total cost (TC), total emission (TE), active power loss (APL), and voltage magnitude deviation (VMD), using a novel constraint handling strategy for OPF incorporating Wind, PV, and PEVs uncertainty. Kyomugisha et al.^28^ applied the mayfly algorithm (MA) to show how well the voltage stability indices performed in the MOOPFs of the power systems. A new and effective improved turbulent flow of water-based optimization (ITFWO) to solve OPF in systems with PV and WT units was introduced in^29^. Mouassa^30^ used the slime mould algorithm (SMA) to control the power flow between various generating resources in order to reduce the main grid's total operating costs. Khorsandi et al.^31^ have applied a modified artificial bee colony (MABC) to solve discrete OPF problems on the IEEE 30-bus and IEEE 118-bus test systems that contain both discrete and continuous variables and take valve point effects (VPE) into account. Elattar et al.^32^, by a modified JAYA (MJAYA) examined two separate test systems, i.e., the IEEE 30-bus system and the IEEE 118-bus system. Saha et al.^33^ used the two‐point estimate method (2PEM) to deal with the unpredictability of renewable generation in OPF. As stated in Table 1, various algorithms have been presented and explored in the literature in relation to the solution to OPFs.

**Table 1:** Summary of the proposed methods for solution of the OPF problems in recent literature.

Reference	The proposed methods	Studied power systems	Objectives
^17^	Moth Swarm Algorithm (MSA)	IEEE 30-bus test system	Operating cost with and without the consideration of prohibited operating zones
^34^	A novel hybrid firefly-bat algorithm with constraints-prior object-fuzzy sorting strategy (HFBA-COFS)	IEEE 30-bus, IEEE 57-bus and IEEE 118-bus systems	Active power loss, total emission and fuel cost
^10^	Sine cosine algorithm (SCA)	Standard 9-bus system	Hydrothermal scheduling (HTS) problem for optimizing fuel cost, emission and combined cost emission
^35^	Improved salp swarm algorithm (ISSA) in compared with moth-flame optimization (MFO), improved harmony search (IHS), genetic algorithm (GA)	IEEE 30-bus, IEEE 57-bus and IEEE 118-bus systems	Minimizing quadratic fuel cost, piecewise quadratic cost, quadratic fuel cost considering the valve-point effect and prohibited zones
^36^	Modified Jaya	IEEE 30-bus and IEEE 118-bus systems	Operational costs, emission, power loss and voltage profile improvement
^33^	Ant lion optimization (ALO)	Modified IEEE 30 bus system	Operational costs, voltage profile, and system-wide transmission power losses
^30^	Bird swarm algorithm (BSA)	IEEE 30 bus system	Total fuel costs, and emissions
^20^	A hybridization of PSO with GWO	Modified IEEE 30 bus test system	Generation costs without and with considering valve point effects, and carbon tax
^19^	Social spider optimization (SSO) algorithms	IEEE 30, IEEE 57 and IEEE 118-bus systems	Fuel cost, power loss, polluted emission, voltage deviation and voltage security index
^18^	Multi-objective ant lion algorithm (MOALA)	IEEE 30-bus, IEEE 57-bus, IEEE 118-bus, IEEE 300-bus systems and on practical Algerian DZ114-bus system	Generation cost, environmental pollution emission, active power losses, and voltage deviation
^16^	Particle swarm optimization (PSO) and shuffle frog leaping algorithms (SFLA)	IEEE 30, IEEE 57 and IEEE 118-bus systems	Power generation involving the prohibit zones, valve point effect and multi-fuel type of generation units, voltage profile, voltage security index, and transmission power losses
^14^	Grey wolf optimizer (GWO) and differential evolution (DE)	IEEE-30 bus and IEEE 118-bus systems	Minimizing the overall fuel cost, the active and reactive power transmission losses, and the voltage security index
^28^	Developed GWO (DGWO)	IEEE 30 bus system	Quadratic fuel cost minimization, piecewise quadratic cost minimization, and quadratic fuel cost minimization considering the valve point effect
^12^	TLBO and genetic algorithm (GA)	19 bus 7336 MW Turkish-wind-thermal power system	Fuel costs for three different loading situations
^15^	Ant lion optimization (ALO)	IEEE 30 and IEEE 57-bus systems	Operating cost, voltage profile, and transmission power losses
^32^	Modified strength Pareto evolutionary algorithm	IEEE 30-bus and IEEE 57-bus systems	Fuel cost and emission
^31^	Multi-objective PSO (MOPSO)	IEEE 30-bus and IEEE 57-bus systems	Generation cost, transmission loss, and the maximum voltage collapse proximity index (VCPI)
^23^	Grey wolf optimizer (GWO)	Modified IEEE-30 and IEEE-57 bus test systems	Generation cost considering renewable energy sources (RES)
^13^	An effective cuckoo search algorithm (ECSA)	IEEE-30 bus system	Minimizing the overall fuel cost, the active power transmission losses, and improve the voltage profile at load buses by minimizing the voltage deviation
^25^	Coronavirus herd immunity optimizer (CHIO), salp swarm algorithm (SSA), and ant lion optimizer (ALO)	IEEE 30-bus and IEEE 57-bus systems	Total fuel costs, emissions level, power losses, voltage deviation, and voltage stability
^21^	Cross entropy-cuckoo search algorithm (CE-CSA)	Modified IEEE 57 bus system	Generation costs with wind energy and solar PV generators and controllable loads
^11^	A modified sine cosine algorithm (MSCA)	IEEE-30 bus and IEEE 118-bus systems	Minimizing the overall fuel cost, the active power transmission losses, and improve the voltage profile at load buses by minimizing the voltage deviation
^29^	Multi-objective glowworm swarm optimization (MOGSO)	Modified IEEE 30 and 300 bus systems	Total generation cost, transmission losses, and voltage stability enhancement index
^28^	A hybrid of a non-dominated sorting genetic algorithm-II (NSGA-II) and fuzzy satisfaction-maximizing method	IEEE 6-units\30-nodes system	Multi-objective dynamic OPF (MDOPF) considering wind generation (WG) and demand response (DR) with fuel cost, carbon emission and active power losses
^27^	Modified moth swarm algorithm (MMSA)	Modified IEEE-30 and IEEE-118 bus test systems	Total fuel costs considering renewable energy sources (RES), power losses, voltage deviation
^24^	Chaotic invasive weed optimization algorithms (CIWOs)	IEEE 30 bus test system	Power generation involving the prohibit zones, valve point effect and multi-fuel type of generation units
^37^	Slime mould algorithm (SMA) in compared with gorilla troops optimizer (GTO), orca predation algorithm (OPA), artificial ecosystem optimizer (AEO), hunger games search (HGS), jellyfish search (JS) optimizer, and success-history based parameter adaptation for DE	IEEE 30-bus test system and Algerian power system, DZA 114-bus	Overall cost of system, including reserve cost for over-estimation and penalty cost for under-estimation of both PV-solar and wind energy
^24^	Success history based adaptive differential evolution (SADE)	Modified IEEE 30 bus system	Generation cost considering renewable energy sources (RES)
^38^	Turbulent flow of water-based optimization (TFWO)	IEEE 30-, 57-bus test system and four large scale power systems called IEEE, 300-bus, 1354pegase, 3012wp, and IEEE 9241pegase power systems	Minimize the fuel cost, emission, active power loss, voltage deviation at the load buses, and voltage stability index (VSI)
^12^	Teaching–learning-based optimization (TLBO) and Lévy TLBO (LTLBO)	IEEE 30-bus and IEEE 57-bus	Minimization of fuel cost without and with valve point loadings, improvement of voltage profile, piecewise quadratic fuel cost functions, and emission

### Contribution and paper organization

OPF minimizes objective function values by finding control variables and equation system values. The technical and economic characteristics of the electrical power system must be considered while choosing this function. Due to the network's development and efficiency needs, system operators always look for fast and efficient ways to operate and design the electric power system. Classical techniques require convex and derivable goal functions and limits to find optimal solutions quickly. Deterministic algorithms get stuck in locally optimal solutions. The OPF problem is a large-scale optimization problem with multiple local and one global optimum in the search space, and also, this is multimodal, non-linear, non-convex, and non-smooth. OPF's nonlinearity traps it in a locally optimal solution. Some methods struggle with continuous and discontinuous data. Classical methods with MOOPF considerations are difficult to design. Heuristic-based optimization algorithms have been developed to overcome this issue. The MOOPF technique may help build power grid operations that best handle optimization concerns. The MOOPF solution seeks the best values for several control variables concerning several objective functions while conforming to equality and inequality constraints. Hence, the goals begin to conflict. Thus, the specified aims become competing in nature.

The concept of abnormal oscillations in water turbulent flow serves as the basis for the turbulent flow of water-based optimization (TFWO) presented in 2021 by Ghasemi et al.^38^. The velocity and direction of this type of turbulent flow constantly fluctuate in a circular pattern. From there, the water spirals downward. In this technique, a whirlpool represents an occurrence in the ocean, river, or sea. Particles are believed to be drawn from the whirlpool’s periphery into the centre. The whirlpool employs the centripetal force of a stream of moving water caused by the ocean tide to exert pressure on them. When applied to a moving object, the centripetal force acts in a circle in a direction perpendicular to and in the same plane as the centre of motion. Because of the centripetal force, the object’s path is changed, but its velocity is unaffected. Many applications have been proposed for TFWO, such as estimating parameters of photovoltaic models^35, 39, 40^, optimal sizing of the energy sources in an isolated hybrid microgrid^41^, and economic load dispatch (ELD) problems^34, 42^.

This work presents a powerful novel algorithm that applies to various problems of optimal load dispatch in hybrid systems by improving the TFWO algorithm. The rest of this paper is organized as follows. In “Formulation of the problem” section, the formulation of the optimal load dispatch problem is discussed. “The proposed algorithm: enhanced TFWO (ETFWO)” section describes the optimization steps of the proposed algorithm. In “Discussions” section, the proposed method has been implemented on the IEEE standard 30-bus network with various load distribution functions, and the results have been analysed. Finally, in “Conclusions” section, the general conclusion of the proposed method is stated.

## Formulation of the problem

### Objective functions

The goal of OPF studies is to determine the constant operational point that minimizes efficiency and minimizes waste. This optimization issue is nonconvex, nonlinear, and massive in scale. OPF can ascertain the current and voltage distributions throughout an electrical grid. Many different OPF formulations and approaches to solving them have been developed in this setting. In addition, modern electricity markets and incorporating of renewable energy sources have contributed to an increase in OPF research.

Many objective functions, which are listed below, are minimized by our proposed algorithm.

#### Energy of wind (EoW) cost function

Wind energy is gradually included in the power system because of its continuously falling cost and emission-free properties. The EoW’s direct cost of electricity can be expressed as in^11^ by:

1 $${C}_{d,w,i}={d}_{w,i}{P}_{w,i}.$$

The operators of wind power facilities are fined if they cannot deliver the volume of energy they had anticipated generating from the wind. ${P}_{w,i}$ is the scheduled power from the same plant, and ${d}_{w,i}$ is the direct cost coefficient associated with the $i$ th wind power plant. There are two components to penalty fees:

1. the underestimation cost that must be included when available wind farm power is not utilized, and
2. the over-estimated cost that is computed for purchasing power from alternative sources (reserves) or load-shedding. It is possible to model these expenditures in the following way^11^:
  2 $${C}_{ue,w,i}={K}_{ue,w,i}{\int }_{{P}_{w,i}}^{{P}_{w,r,i}}\left({P}_{i}-{P}_{w,i}\right)f\left({P}_{i}\right)d{P}_{i},$$
  3 $${C}_{oe,w,i}={K}_{oe,w,i}{\int }_{0}^{{P}_{w,i}}\left({P}_{w,i}-{P}_{i}\right)f\left({P}_{i}\right)d{P}_{i},$$
  where $i$ is from the set $\{1, 2,\dots , {n}_{w}\}$, the ${n}_{w}$ is the number of the wind power plants. The PDF, i.e., the probability density function of EoW output power can be represented by $f\left({P}_{i}\right)$ for $i$ th wind power plant. And, ${K}_{oe,w,i}$ is the reserve cost coefficient pertaining to the $i$ th wind power plant. Also, ${K}_{ue,w,i}$ is the penalty cost coefficient for the $i$ th wind power plant, ${P}_{w,r,i}$ is rated output power from the same windfarm.

The following cost function describes the overall expenditures for EoW:

4 $$\sum_{i=1}^{{n}_{w}}COS{T}_{w,i}=\sum \limits _{i=1}^{{n}_{w}}\left({C}_{d,w,i}+{C}_{ue,w,i}+{C}_{oe,w,i}\right),$$

where ${C}_{ue,w,i}$ is the reserve cost for the $i$ th wind plant, ${C}_{oe,w,i}$ is the penalty cost of wind power surplus.

We employ the Weibull distribution to describe the randomness of wind speed. Hence the probability density function (PDF) $f\left({V}_{w}\right)$ and the cumulative distribution function (CDF) $F\left({V}_{w}\right)$ can be defined as in^6^ by for the $K$ th solar EoW plant:

5 $$f\left({V}_{w}\right)=\frac{K}{C}{\left(\frac{{V}_{w}}{C}\right)}^{K-1}{e}^{-{\left(\frac{{V}_{w}}{C}\right)}^{K}},{V}_{w}>0,$$

6 $$F\left({V}_{w}\right)=1-{e}^{-{\left(\frac{{V}_{w}}{C}\right)}^{k}},{V}_{w}>0.$$

Calculating the energy that EoW produces entails:

7 $${P}_{w}\left({V}_{w}\right)=\left\{\begin{array}{ll}0, & \quad {V}_{w,out}<{V}_{w}<{V}_{w,in},\\ \frac{{P}_{w,r}}{{V}_{w,r}-{V}_{w,in}}{(V}_{w}-{V}_{w,in}), & \quad {V}_{w,in}\le {V}_{w}\le {V}_{w,r},\\ {P}_{w,r},&\quad {V}_{w,r}<{V}_{w}<{V}_{w,out},\end{array}\right.$$

where ${V}_{w}$, ${V}_{w,r}$, ${V}_{w,in}$, and ${V}_{w,out}$ represent the speed, the rated speed of EoW generators, the cut-in speed, and the cut-out speed, respectively. Moreover, the contour and scaling variables of the Weibull distribution are shown by $K$ and $C$, respectively.

#### PV cost function

Because of their low price and easy implementation, photovoltaic (PV) systems are becoming increasingly popular as a sustainable energy option. Predicting PV’s power output is challenging because its features rely highly on a wide range of variables. Following is a breakdown of how to figure out how much PV power will cost to generate ${C}_{ue,pv,i}$ and how much that will cost in penalties ${C}_{oe,pv,i}$^11^:

8 $${C}_{d,pv,i}={d}_{pv,i}{P}_{\mathit{pv},i},$$

9 $${C}_{ue,pv,i}={K}_{ue,pv,i}{\int }_{{P}_{pv,i}}^{{P}_{pv,r,i}}\left(P-{P}_{pv,i}\right)f\left(P\right)dP,$$

10 $${C}_{oe,pv,i}={K}_{oe,pv,i}{\int }_{0}^{{P}_{pv,i}}\left({P}_{pv,i}-P\right)f\left(P\right)dP,$$

where ${d}_{pv,i}$ is the direct cost coefficient associated with the $i$th solar PV plant, ${P}_{\mathit{pv},i}$ is the scheduled power from the same plant. $i = 1, . . . ,{n}_{v}$ and $f\left(P\right)$ represent the PDF of the PV unit’s output power.${n}_{v}$ shows the number of the PV plants.

Using this method, we may calculate the full price of photovoltaics.

11 $$\sum_{i=1}^{{n}_{v}}COS{T}_{pv,i}=\sum \limits _{i=1}^{{n}_{v}}\left({{C}_{d,pv,i}+C}_{ue,pv,i}+{C}_{oe,pv,i}\right).$$

To calculate the PDF of the $i$th PVs’ output power, one can use the following: photovoltaic (PV) cells, also known as solar cells, are susceptible to sunlight. When modeling the probability density function (PDF) of solar radiation $f\left(R\right)$, a beta distribution is a good fit^11^:

12 $$f\left(R\right)=\frac{\Gamma \left(\alpha +\beta \right)}{\Gamma \left(\alpha \right)\Gamma \left(\beta \right)}{R}^{\alpha -1}{\left(1-R\right)}^{\beta },$$

where $\Gamma$, is the gamma function and $\alpha , \beta$ are parameters of the beta distribution. Moreover, the solar radiation is denoted by $R$.

The following equation can be used to determine the relationship between the power output of PV and the power output of the solar cell generator, which is connected to solar radiation^11^:

13 $${P}_{pv}\left(R\right)=\left\{\begin{array}{ll}{P}_{pv,r}\left(\frac{{R}^{2}}{{R}_{C}{R}_{STD}}\right), & \quad 0\le R\le {R}_{C},\\ {P}_{pv,r}\left(\frac{R}{{R}_{STD}}\right),& \quad {R}_{C}\le R\le {R}_{STD},\\ {P}_{pv,r},& \quad {R}_{STD}\le R,\end{array}\right.$$

where ${R}_{C}$ is solar radiation in W/m^2^ and ${R}_{STD}$ is the rate of solar radiation transfer to the earth. A standard solar radiation point is typically established at 150 W/m^2^ and normal conditions call for 100 W/m^2^.

#### Basic fuel cost function

Primarily, OPF is used to reduce the cost of essential gasoline. The fuel cost of a power plant is typically described as a quadratic function^42^:

14 $$J=\sum \limits_{i=1}^{{n}_{G}}{f}_{i}({P}_{Gi})=\sum \limits _{i=1}^{{n}_{G}}({a}_{i}+{b}_{i}{P}_{Gi}+{c}_{i}{P}_{Gi}^{2}),$$

where $i$ and ${n}_{G}$ represent the $i$th power plant and the number of power plants, respectively. Furthermore, the coefficients ${a}_{i}$, ${b}_{i}$, and ${c}_{i}$ represents the cost coefficients for the $i$th power plant and ${P}_{Gi}$ is power of the $i$th power plant.

#### Piecewise quadratic fuel cost function

Typically, power plants will utilize the lowest fuel available for a given operating range.

Piecewise quadratic is the form of the fuel cost function for such a setup.

15 $$J=\sum \limits_{i=1}^{{n}_{G}}{f}_{i}({P}_{Gi}).$$

The following function can be used to figure out the cost of each quadratic component of fossil fuels^42^:

16 $${f}_{i}\left({P}_{Gi}\right)=\sum \limits_{k=1}^{{n}_{f}}\left({a}_{i,k}+{b}_{i,k}{P}_{Gi}+{c}_{i,k}{P}_{Gi}^{2}\right),$$

where ${n}_{f}$ is the number of fossil fuel options for the $i$th power plant. Moreover, the coefficients ${a}_{i,k}$, ${b}_{i,k}$, ${c}_{i,k}$ are costs of the $i$th power plant for the $k$th fuel option.

#### Piecewise quadratic fuel cost with valve point loading

Generator cost is a cumulative heat rate graph that is convex but is interrupted by the entrance level valves in large turbines. For each generator’s true cost to be determined, the valve point impact must be included in^42^:

17 $$J= \sum \limits_{i=1}^{{n}_{G}}\left({a}_{i}+{b}_{i}{P}_{Gi}+{c}_{i}{P}_{Gi}^{2}+\left|{e}_{i}\mathrm{sin}\left({f}_{i}\left({P}_{Gi}^{min}-{P}_{Gi}\right)\right)\right|\right),$$

where coefficients ${e}_{i}$ and ${f}_{i}$ represent valve point cost of the $i$th power plant.

#### Emission cost function

Power plants that rely on fossil fuels, such as petroleum, coal, and natural gas, burn these materials to generate electricity. When something is burned, a lot of pollution is released. The sum of all forms of emission taken into account, such as SOX and NOX, with appropriate pricing or weighting on each pollutant emitted, can be used to represent the minimization of emission function for OPF problems. In this study, we use the following function to represent NOx and SOx emissions, two significant air pollutants^42^:

18 $$\sum \limits_{i=1}^{{n}_{G}}{f}_{Ei}({P}_{Gi})=\sum \limits _{i=1}^{{n}_{G}}\left({\alpha }_{i}+{\beta }_{i}{P}_{Gi}+{\gamma }_{i}{P}_{Gi}^{2}+{\xi }_{i}\mathrm{exp}({\theta }_{i}{P}_{Gi})\right).$$

The emission coefficients of the $i$th power plant are shown by ${\alpha }_{i}$ (ton/h), ${\beta }_{i}$ (ton/h MW), ${\gamma }_{i}$ (ton/h MW2), ${\xi }_{i}$ (ton/h) and ${\theta }_{i}$ (1/MW).

#### Power loss cost function

The generation of electricity and the global demand for it are inversely correlated. Therefore, limiting energy loss is crucial for OPF issues. Power systems inevitably have transmission losses because of the resistance of the transmission cables. Transmission systems will inevitably experience power losses. The majority of active power loss happens when the power system is in use. The cost of energy production directly relates to the power loss. The next power loss function must be satisfied in order to decrease transmission lines’ active power loss^43^:

19 $${P}_{Loss}=\sum \limits_{i=1}^{{N}_{l}}\sum \limits_{\begin{array}{c}j=1\\ j\ne i\end{array}}^{{N}_{l}}\left({G}_{ij}{V}_{i}^{2}+{B}_{ij}{V}_{j}^{2}-2{V}_{i}{V}_{j}\mathrm{cos}{\delta }_{ij}\right).$$

All parameters mentioned in Eq. (19) are defined in^44^.

#### Voltage deviation (VD) cost function

One of the most significant security and service indicators is bus voltage. In an OPF problem, considering simply a cost-based objective will lead to a workable solution with an unacceptable voltage profile. Consequently, a dual goal function is required to reduce fuel costs and enhance voltage profiles by reducing load bus voltage deviations from 1.0 per unit to provide a desired voltage profile^42^:

20 $$VD=\sum \limits_{i=1}^{{n}_{PQ}}\left|V{L}_{i}-1\right|,$$

where ${n}_{PQ}$ and $V{L}_{i}$ are the numbers of load bus bars and the $i$th voltage of load buses, respectively.

### Constraints

The following are some requirements that the OPF optimization issue must meet^42^:

1. Active and reactive power balances
  21 $${P}_{Gi}-{P}_{Di}=\sum \limits_{j=1}^{n}{V}_{i}{V}_{j}\left({G}_{ij}\mathrm{cos}{\delta }_{ij}+ {B}_{ij}\mathrm{sin}{\delta }_{ij}\right), \quad i=1,...,n,$$
  22 $${Q}_{Gi}-{Q}_{Di}=\sum \limits_{j=1}^{n}{V}_{i}{V}_{j}\left[{G}_{ij}\mathrm{cos}{\delta }_{ij}+ {B}_{ij}\mathrm{sin}{\delta }_{ij}\right], \quad i=1,...,n,$$
  where ${P}_{Gi}$, ${Q}_{Gi}$, ${P}_{Di}$, and ${Q}_{Di}$ are the active and reactive power generations and load demands at the $i$th bus, respectively.
2. The power plant voltage magnitude
  23 $${V}_{i}^{\mathrm{min}}\le {V}_{i}\le {V}_{i}^{\mathrm{max}}, \quad i=\mathrm{1,2},\dots ,{n}_{G},$$
  where ${V}_{i}^{\mathrm{min}}$ and ${V}_{i}^{\mathrm{max}}$ are minimum and maximum bound of the $i$th bus voltage of power plants ${V}_{i}$.
3. Active and reactive power
  24 $${P}_{Gi}^{\mathrm{min}}\le {P}_{Gi}\le {P}_{Gi}^{\mathrm{max}}, \quad i=\mathrm{1,2},\dots ,{n}_{G},$$
  25 $${Q}_{Gi}^{\mathrm{min}}\le {Q}_{Gi}\le {Q}_{Gi}^{\mathrm{max}}, \quad i=\mathrm{1,2},\dots ,{n}_{G},$$
  where ${P}_{Gi}^{\mathrm{min}}$ and ${P}_{Gi}^{\mathrm{max}}$ are active power margins of the $i$th generator, and ${Q}_{Gi}^{\mathrm{min}}$ and ${Q}_{Gi}^{\mathrm{max}}$ are borders of reactive power of the $i$th traditional generator.
4. Transformer tap
  26 $${T}_{i}^{\mathrm{min}}\le {T}_{i}\le {T}_{i}^{\mathrm{max}}, \quad i=\mathrm{1,2},\dots ,{N}_{tab},$$
  ${T}_{i}^{\mathrm{min}}$ and ${T}_{i}^{\mathrm{max}}$ are boundaries of the $i$th tap changer transformer ${T}_{i}$, ${N}_{tab}$ is the number of tap changer to the network.
5. Shunt compensator
  27 $${N}_{i}^{\mathrm{min}}\le {N}_{i}\le {N}_{i}^{\mathrm{max}}, \quad i=\mathrm{1,2},\dots ,{N}_{cap},$$
  where the number of capacitors linked to the network is represented by ${N}_{cap}$.
6. Transmission line loading
  28 $$\left|{S}_{i}\right|\le {S}_{i}^{max}, \quad i=\mathrm{1,2},\dots ,{N}_{l},$$
  where ${S}_{i}^{max}$ is MVA’s maximum and ${N}_{l}$ is the number of lines.
7. Active power of EoW
  29 $$0\le {P}_{w,i}\le {P}_{w,r,i}.$$
8. Photovoltaic active power
  30 $$0\le {P}_{pv,i}\le {P}_{pv,r,i}.$$

### Constraints control

To consider the violation of the constraints of a penalty function, the following term is considered in the basic objective function:

31 $$\begin{aligned} J&=\sum \limits _{i=1}^{{n}_{G}}{f}_{i}({P}_{Gi})+{\lambda }_{P}\cdot {\left({P}_{G1}-{P}_{G1}^{\mathrm{lim}}\right)}^{2}+{\lambda }_{V}\sum \limits _{i=1}^{{n}_{PQ}}{\left({VL}_{i}-{VL}_{i}^{\mathrm{lim}}\right)}^{2} \\ & \quad +{\lambda }_{Q}\sum \limits _{i=1}^{{n}_{G}}{\left({Q}_{Gi}-{Q}_{Gi}^{\mathrm{lim}}\right)}^{2}+{\lambda }_{S}\sum \limits _{i=1}^{{N}_{l}}{\left({S}_{i}-{S}_{i}^{\mathrm{lim}}\right)}^{2}, \end{aligned}$$

Equation (31) is the general objective function of optimal load dispatch, in which all kinds of problem constraints are applied In this equation, part ${\sum }_{i=1}^{{n}_{G}}{f}_{i}({P}_{Gi})$ is the general objective function that ${P}_{Gi}$ can be different types of power generation sources, which in this article can include thermal, wind, or solar generators. The rest of Eq. (31) is the part added to consider the constraints of the load dispatch problem, i.e.:

$${\lambda }_{P}\cdot {\left({P}_{G1}-{P}_{G1}^{\mathrm{lim}}\right)}^{2}+{\lambda }_{V}\sum \limits _{i=1}^{{n}_{PQ}}{\left({VL}_{i}-{VL}_{i}^{\mathrm{lim}}\right)}^{2}+{\lambda }_{Q}\sum \limits _{i=1}^{{n}_{G}}{\left({Q}_{Gi}-{Q}_{Gi}^{\mathrm{lim}}\right)}^{2}+{\lambda }_{S}\sum \limits _{i=1}^{{N}_{l}}{\left({S}_{i}-{S}_{i}^{\mathrm{lim}}\right)}^{2}$$

In this paper, the penalty factors symbolized by ${\lambda }_{P}$, ${\lambda }_{V}$, ${\lambda }_{Q},$ and ${\lambda }_{S}$, which have been chosen to have large positive values, have been set to 10,000,000. Furthermore, ‘limit values’ ${P}_{G1}^{\mathrm{lim}}$, ${VL}_{i}^{\mathrm{lim}},$ ${Q}_{Gi}^{\mathrm{lim}}$, and ${S}_{i}^{\mathrm{lim}}$ in Eq. (31) are variables that are defined by the following equation:

32 $${x}^{\mathrm{lim}}=\left\{\begin{array}{ll}x,& \quad {x}^{\mathrm{min}}\le x\le {x}^{\mathrm{max}},\\ {x}^{\mathrm{max}},& \quad x>{x}^{\mathrm{max}},\\ {x}^{\mathrm{min}},& \quad x<{x}^{\mathrm{min}}.\end{array}\right.$$

## The proposed algorithm: enhanced TFWO (ETFWO)

### Turbulent flow of water-based optimization (TFWO)

Following the above mentioned introductory paragraphs, we present and simulate the various stages of the original TFWO algorithm’s implementation and performance.

#### Formation of whirlpools

The algorithm begins by random partitioning its initial population ${X}^{0},$ that consist from ${N}_{pop}$ members, into ${N}_{Wh}$ whirlpool sets ${S}_{{Wh}_{j}}, j=1,...,{N}_{Wh},$ of the same cardinality (thus, some disjunct subsets of the set ${X}^{0}$ of the same cardinality $\lceil{N}_{pop}/{N}_{Wh}\rceil$). Next, a member, denoted ${Wh}_{j},$ with the best fitness value $f ({Wh}_{j})$, is selected in each whirlpool set ${S}_{{Wh}_{j}}$ and it is called as the whirlpool of the whirlpool set ${S}_{{Wh}_{j}}$ or the center of the set ${S}_{{Wh}_{j}}$ (or hole of ${S}_{{Wh}_{j}}$). This whirlpool ${Wh}_{j}$ pulls the other objects in the $j$th whirlpool set ${S}_{{Wh}_{j}}, j=1,...,{N}_{Wh}.$

#### The whirlpools effects on objects

**Figure 1:**
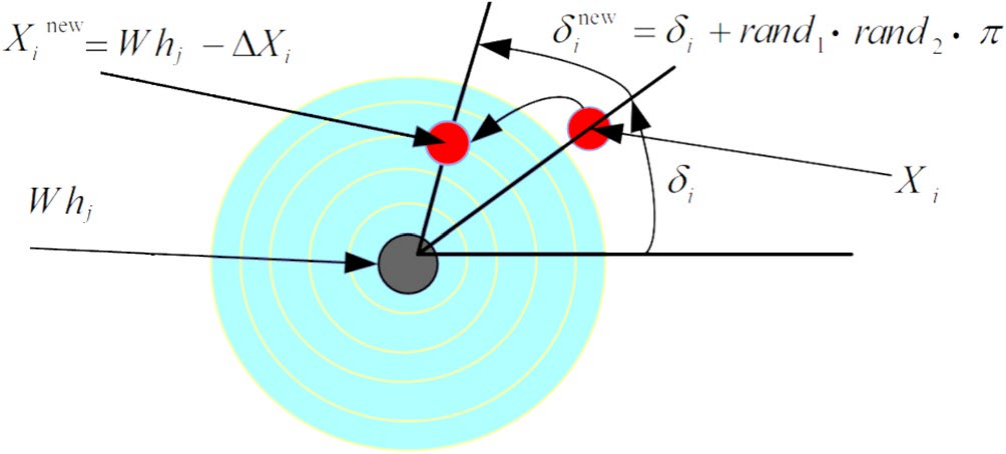
An optimization model by the whirlpool ${Wh}_{j}$ (Ghasemi^38^).

Every whirlpool ${Wh}_{j}, j=1,...,{N}_{Wh},$ serves as a drinking hole and tends to apply a centripetal force on the other surrounding objects in the set ${S}_{{Wh}_{j}}$ to bring them into alignment with ${Wh}_{j}$. Because of this, the $j$th whirlpool ${Wh}_{j}$, $j=1,...,{N}_{Wh},$ with its local position in ${S}_{{Wh}_{j}},$ behaves in a way that its location is merged with that of the $i$th object ${X}_{i}$ from ${S}_{{Wh}_{j}}$, i.e., ${X}_{i} = {Wh}_{j}$. Furthermore, we will also assume that individual whirlpools will also influence each other. Thus, whirlpools based on at mutual distances $Wh-{Wh}_{j}$ between them and cost merits $f (\cdot )$ create various differences $\Delta {X}_{i}$. Therefore, the new location of the $i$th particle is ${X}_{i}^{new} = {Wh}_{j} - \Delta {X}_{i}$. Figure 1 depicts the impacts of a whirlpool on the objects in its whirlpool set. In Fig. 1 we can see that the object ${X}_{i}$ is moving at an angle ${\delta }_{i}$ around and toward its whirlpool ${Wh}_{j}$. Therefore, this angle fluctuates at each iteration of the algorithm, which can be indicated as ${\delta }_{i}^{\text{new}}={\delta }_{i}+ran{d}_{1}\cdot ran{d}_{2}\cdot \pi$.

By determining the strongest and weakest whirlpools (thus, whirlpools with the lesser and greater weighted distance to all objects) by Eq. (33). Hence, we can compute using Eqs. (34) and (35) the new position of the $i$th object ${X}_{i}^{new}$.

33 $${\Delta }_{t}=f\left({Wh}_{t}\right)\cdot \sqrt{\left|W{h}_{t}-\sum_{j}W{h}_{j}\right|},$$

34 $$\Delta {X}_{i}=\left(1+\left|\mathrm{cos}{\delta }_{i}^{\text{new}}-\mathrm{sin}{\delta }_{i}^{\text{new}}\right|\right)\cdot \left(\mathrm{cos}{\delta }_{i}^{\text{new}}\cdot rand\left(1,D\right)\cdot \left(W{h}_{f}-{X}_{i}\right)-\mathrm{sin}{\delta }_{i}^{\text{new}}\cdot \mathrm{rand}\left(1,D\right)\cdot \left(W{h}_{w}-{X}_{i}\right)\right),$$

35 $${X}_{i}^{new} = {Wh}_{j} - \Delta {X}_{i},$$

where $Wh$ with minimum and maximum values of ${\Delta }_{t}$ are denoted by $W{h}_{f}$ and $W{h}_{w}$, respectively, and ${\delta }_{i}^{\text{new}}$ is the new angle of the $i$th object. Pseudocodes 1 and 2 show the proposed mathematical model for updating the position of the $i$th object:

#### Pseudo-code 1

$$\mathrm{for} \,\,\,t=1:{N}_{Wh}$$

${\Delta }_{t}=f\left({Wh}_{t}\right)\cdot \sqrt{\left|{Wh}_{t}-\mathrm{sum}\left({X}_{i}\right)\right|}$ // Where the function sum(A) returns the sum of all elements of the vector A.

$$\mathrm{end \; for};$$

$${Wh}_{f}=Wh\;\mathrm{ with \; the \; minimum \;value \;of \;}{\Delta }_{t};$$

$$;{Wh}_{w}=Wh\;\mathrm{ with \; the \; maximum\; value \;of\; }{\Delta }_{t};$$

$${\delta }_{i}^{\text{new}}={\delta }_{i}+ran{d}_{1}\cdot ran{d}_{2}\cdot \pi ;$$

$$\Delta {X}_{i}=\left(1+\left|\mathrm{cos}{\delta }_{i}^{\text{new}}-\mathrm{sin}{\delta }_{i}^{\text{new}}\right|\right)\cdot \left(\mathrm{cos}{\delta }_{i}^{\text{new}}\cdot rand(1,D)\cdot \left(W{h}_{f}-{X}_{i}\right)-\mathrm{sin}{\delta }_{i}^{\text{new}}\cdot rand(1,D)\cdot \left(W{h}_{w}-{X}_{i}\right)\right);$$

$${X}_{i}^{new} = {Wh}_{j} - \Delta {X}_{i}$$

#### Pseudo-code 2

$${X}_{i}^{new}=\mathrm{min}\left(\mathrm{max}\left({X}_{i}^{new},{X}^{\mathrm{min}}\right),{X}^{\mathrm{max}}\right) ;$$

$$\mathrm{if} \; f\left({X}_{i}^{new}\right)\le f\left({X}_{i}\right) \; \mathrm{then } \;{X}_{i}={X}_{i}^{new}; f\left( {X}_{i}\right)=f\left({X}_{i}^{new}\right)$$

$$\mathrm{end \, if}.$$

#### Centrifugal force

**Figure 2:**
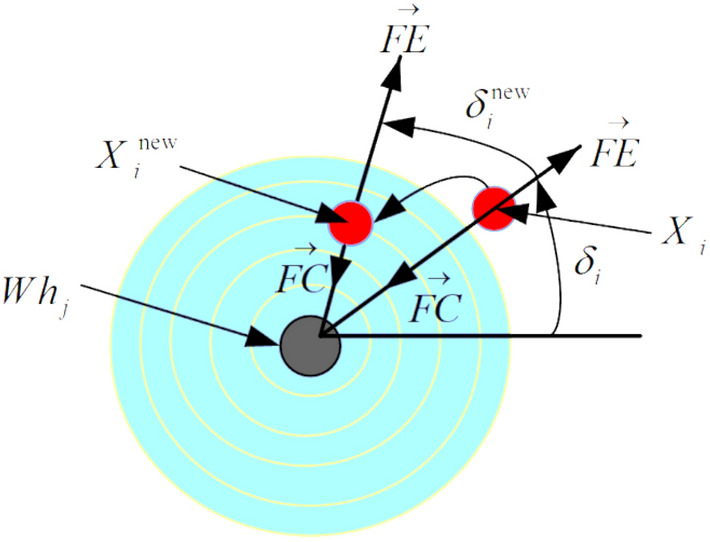
The various types of forces in a whirlpool ${Wh}_{j}$ (Ghasemi^38^).

Sometimes the centripetal or traction force of the whirlpool is defeated by the centrifugal force ${FE}_{i}$, and the object is arbitrarily transferred to a new site. To account for the unpredictable nature of the centrifugal force experienced by each object, there is a random variable along a single dimension of the objectives’ space (or the solution). To do this, we first determine the centrifugal force according to the angle it makes with the center of the hole by Eq. (36) and if this force is greater than a random value $r$ from the interval $\left[\mathrm{0,1}\right]$, we conduct the centrifugal action for the chosen the $p$th dimension of the $i$th object using Eq. (37). Pseudo-code 3 is described in Fig. 2.

36 $${FE}_{i}=\frac{{\mathrm{sin}}^{4}\left(2{\delta }_{i}^{\mathrm{new}}\right)}{16},$$

37 $${x}_{i,p}={x}_{p}^{\mathrm{max}}-{x}_{i,p}+{x}_{p}^{\mathrm{min}}.$$

#### Pseudo-code 3

$${FE}_{i}=\frac{{\mathrm{sin}}^{4}\left(2{\delta }_{i}^{\mathrm{new}}\right)}{16}$$

$$\mathrm{if}\,\, \,r<{FE}_{i}\,\, \mathrm{then }\,\,p=\mathrm{round} \left(1+r\cdot \left(D-1\right)\right)$$

$${x}_{i,p}={x}_{p}^{\mathrm{max}}-{x}_{i,p}+{x}_{p}^{\mathrm{min}}; f\left( {X}_{i}\right)=f\left({X}_{i}^{new}\right)$$

$$\mathrm{end\; if}.$$

#### Interactions between the whirlpools

Whirlpools interact and move one another, much like how they affect the objects nearby. We somehow approximated this phenomenon to be similar to the effects of whirlpools on objects and particles, where each whirlpool has a tendency to draw other whirlpools, exert centripetal force on them, and cause them to fall into their holes. Using the objective function of the nearest whirlpool and the minimum quantity from Eq. (38), we can model and compute ${\Delta Wh}_{j}$. Then, the variation of the position of the $j$th whirlpool to decrease in its objective function is stated by Eqs. (39) and (40), where ${\delta }_{j}$ is the $j$th whirlpool’s angle.

38 $${\Delta }_{t}=\left|W{h}_{t}-\sum_{j}W{h}_{j}\right|\cdot f\left(W{h}_{t}\right),$$

39 $$\Delta W{h}_{j}=\mathrm{rand}\left(1,D\right)\cdot \left(W{h}_{f}-W{h}_{j}\right)\cdot \left|\mathrm{cos }{\delta }_{j}^{\text{new}}+\mathrm{sin}{\delta }_{j}^{\text{new}}\right|,$$

40 $$W{h}_{j}^{\text{new}}=W{h}_{f}-\Delta W{h}_{j}.$$

#### Pseudo-code 4

$$\mathrm{for} \;t=1:{N}_{Wh}$$

$${\Delta }_{t}=\left|W{h}_{t}-\mathrm{sum}\left(W{h}_{j}\right)\right|\cdot f\left(W{h}_{t}\right)$$

$$\mathrm{end \;for };$$

$${Wh}_{f}=Wh\;\mathrm{ with\; the\; minimum\; value} \;\mathrm{of} \;{\Delta }_{t};$$

$${Wh}_{j}^{\mathrm{new}}={Wh}_{f}-\Delta {Wh}_{j};$$

$$\Delta W{h}_{j}=\mathrm{rand}\left(1,D\right)\cdot \left(W{h}_{f}-W{h}_{j}\right)\cdot \left|\mathrm{cos }{\delta }_{j}^{\text{new}}+\mathrm{sin}{\delta }_{j}^{\text{new}}\right| ;$$

$${\delta }_{j}^{\text{new}}={\delta }_{j}+ran{d}_{1}\cdot ran{d}_{2}\cdot \pi .$$

#### Pseudo-code 5

$${Wh}_{j}^{new}=\mathrm{min}\left(\mathrm{max}\left({Wh}_{j}^{new},{X}^{\mathrm{min}}\right),{X}^{\mathrm{max}}\right)$$

$$\mathrm{if}\,\, f\left({Wh}_{j}^{new}\right)\le f\left({Wh}_{j}\right) \mathrm{then}$$

$${Wh}_{j}={Wh}_{j}^{new}; f\left({Wh}_{j}\right)= f\left({Wh}_{j}^{new}\right)$$

$$\mathrm{end\; if}.$$

If the strongest new member of the whirlpool’s set has more strength, i.e., lower goal function value than the center, it becomes the new center for the following iteration. As a result, the new members’ rules are substituted with the whirlpool’s prior center, i.e.,

#### Pseudo-code 6

$$\mathrm{if}\,\, f\left({X}^{best}\right)\le f\left({Wh}_{j}\right) \mathrm{then}$$

$$Wh_{j} \leftrightarrow X^{best} ;\;f\left( {Wh_{j} } \right) \leftrightarrow f\left( {X^{best} } \right)$$

end if.

### Enhanced TFWO (ETFWO)

**Figure 3:**
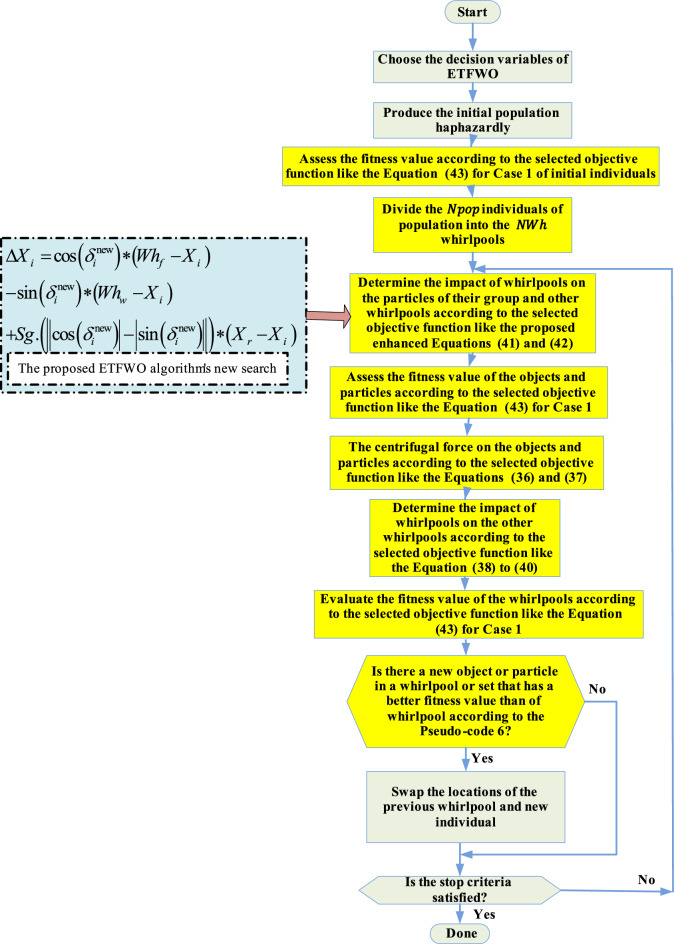
The flowchart of the suggested ETFWO algorithm.

TFWO has poor solution accuracy, slow convergence speed, and immature behavior when solving complex optimization problems. In this research, a new ETFWO algorithm is developed to improve TFWO’s weak points, thereby facilitating information exchange between individuals. Because each participant in the population interacts more frequently, the search can advance more precisely in the search area while ignoring local optimal solutions. This method significantly improves the TFWO algorithm’s performance by allowing it to explore the search space more effectively and increasing its ability to exploit. The proposed ETFWO algorithm's new search is described in Eq. (41). In this equation, the local search, ${X}_{r}-{X}_{i}$, is added in an effective way to the search equation of members in the main TFWO algorithm. This new average has a big effect on how well the proposed algorithm searches locally and, as a result, solves problems in general.

41 $$\Delta {X}_{i}=\mathrm{cos }\left({\delta }_{i}^{\text{new}}\right)\left({Wh}_{f}-{X}_{i}\right)-\mathrm{sin}\left({\delta }_{i}^{\text{new}}\right)\left({Wh}_{w}-{X}_{i}\right)+\mathrm{Sg}\cdot \left|\left|\mathrm{cos }\left({\delta }_{i}^{\text{new}}\right)\right|-\left|\mathrm{sin}\left({\delta }_{i}^{\text{new}}\right)\right|\right|\cdot \left({X}_{r}-{X}_{i}\right),$$

where $$\mathrm{Sg}=\left\{\begin{array}{cc}1,& f\left({X}_{r}\right)\le f\left({X}_{i}\right),\\ -1,& f\left({X}_{r}\right)>f\left({X}_{i}\right),\end{array}\right.$$

42 $${X}_{i}^{\text{new}}=W{h}_{j}-\Delta {X}_{i},$$

where ${X}_{r}$ is a random member selected haphazardly from the entire population. The flowchart of the proposed ETFWO is provided in Fig. 3.

### ETFWO for different OPF problems in the IEEE 30 bus test system

In eight cases of OPF, the TFWO and ETFWO have been implemented on the IEEE 30 bus test system. For both the TFWO and ETFWO was used ${N}_{pop}=30$ and the maximum number of iterations set as 400. In^42^, we can find information about the power systems’ parameters.

### OPF solutions IEEE 30-bus network

**Figure 4:**
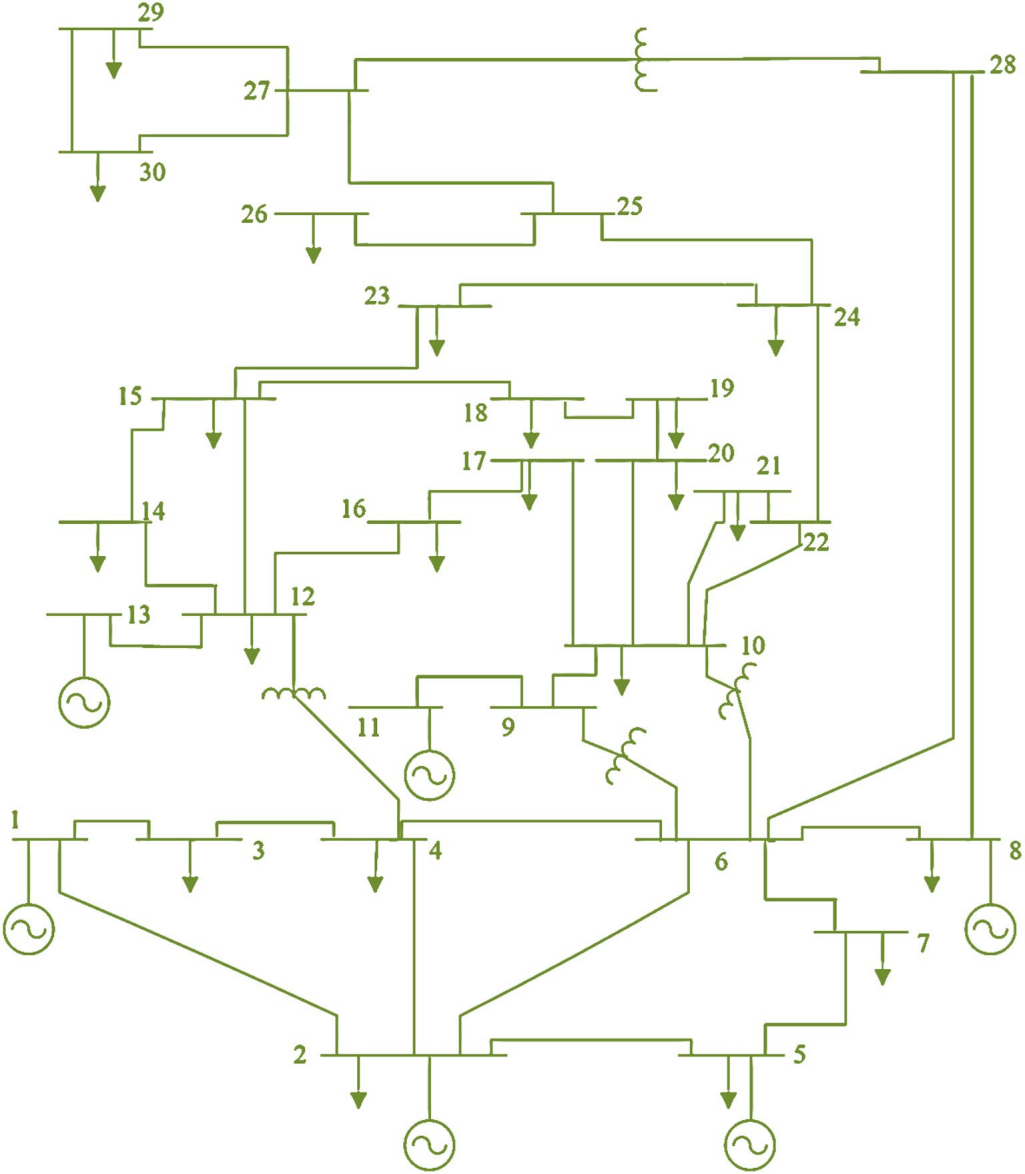
The layout of IEEE 30-bus network.

As shown in Fig. 4, see^42^, four transformers with non-nominal tap ratios are situated on lines 6–9, 6–10, 4–12, and 28–27, whereas six generators are placed on buses 1, 2, 5, 8, 11, and 13. The overall network demand at a base capacity of 100 MVA is 2.834 p.u. The highest and lowest voltages of each load bus were adjusted to 1.05 and 0.95 p.u., respectively.

All of the final ETFWO results for the six OPF’s scenarios without WT and PV producers of the 30-bus power system are presented in Table 2.

**Table 2:** The optimal variables for OPF without stochastic renewable energy obtained by ETFWO.

Variables	Cases					
	1	2	3	4	5	6
*P*_*G1*_	177.1267	140.0000	198.7354	102.6144	176.3648	122.2616
*P*_*G2*_	48.7082	55.0000	44.9265	55.5551	48.7500	52.4737
*P*_*G5*_	21.3899	24.0236	18.4259	38.1132	21.7409	31.4714
*P*_*G8*_	21.2594	34.9999	10.0000	35.0000	22.1242	35.0000
*P*_*G11*_	11.9355	18.5330	10.0000	30.0000	12.2883	26.7304
*P*_*G13*_	12.0000	17.5860	12.0000	26.6468	12.0012	21.0542
*V*_*G1*_	1.0836	1.0730	1.0818	1.0698	1.0427	1.0729
*V*_*G2*_	1.0605	1.0564	1.0582	1.0576	1.0230	1.0571
*V*_*G5*_	1.0337	1.0303	1.0310	1.0359	1.0155	1.0318
*V*_*G8*_	1.0382	1.0401	1.0372	1.0438	1.0073	1.0408
*V*_*G11*_	1.0991	1.0912	1.0978	1.0835	1.0469	1.0380
*V*_*G13*_	1.0519	1.0627	1.0629	1.0573	0.9874	1.0255
*T*_*6–9*_	1.0711	1.0417	1.0450	1.0653	1.0680	1.0970
*T*_*6–10*_	0.9185	0.9273	0.9701	0.9205	0.9000	0.9498
*T*_*4-12*_	0.9775	1.0061	0.9935	0.9901	0.9413	1.0354
*T*_*28–27*_	0.9736	0.9725	0.9785	0.9750	0.9709	1.0049
*Q*_*C10*_	2.7850	0.2033	4.8040	4.9151	4.9299	2.9129
*Q*_*C12*_	1.2070	4.1394	1.1443	0.1626	1.5533	0.2195
*Q*_*C15*_	4.4206	4.4354	4.5239	4.4632	4.9995	3.8386
*Q*_*C17*_	5.0000	4.9998	5.0000	5.0000	0.0002	5.0000
*Q*_*C20*_	4.2462	4.2974	4.3420	4.2446	4.9990	4.9912
*Q*_*C21*_	5.0000	4.9999	5.0000	5.0000	4.9981	5.0000
*Q*_*C23*_	3.2809	3.2116	3.3027	3.2604	4.9955	4.3400
*Q*_*C24*_	5.0000	5.0000	5.0000	5.0000	4.9983	5.0000
*Q*_*C29*_	2.6430	2.5880	2.6571	2.5550	2.6827	2.6548
Cost ($/h)	800.4792	646.4860	832.1625	859.0080	803.7868	830.2325
Emission (t/h)	0.3662	0.2835	0.4378	0.2289	0.3638	0.2530
Power losses (MW)	9.0197	6.7425	10.6878	4.5295	9.8694	5.5913
V.D. (p.u.)	0.9085	0.9235	0.8584	0.9328	0.0945	0.2962

#### Case 1: Minimizing fuel costs

This objective function takes into account reducing the overall fuel cost of producing electricity, which is represented by the quadratic cost curve shown below.

43 $$\begin{aligned}{J}_{1} &=\sum \limits _{i=1}^{{n}_{G}}\left({a}_{i}+{b}_{i}{P}_{Gi}+{c}_{i}{P}_{Gi}^{2}\right)+{\lambda }_{P}{\cdot \left({P}_{G1}-{P}_{G1}^{\mathrm{lim}}\right)}^{2}+{\lambda }_{V}\sum \limits _{i=1}^{{n}_{PQ}}{\left({VL}_{i}-{VL}_{i}^{\mathrm{lim}}\right)}^{2} \\ & \quad +{\lambda }_{Q}\sum \limits _{i=1}^{{n}_{G}}{\left({Q}_{Gi}-{Q}_{Gi}^{\mathrm{lim}}\right)}^{2}+{\lambda }_{S}\sum \limits _{i=1}^{{N}_{l}}{\left({S}_{i}-{S}_{i}^{\mathrm{lim}}\right)}^{2},\end{aligned}$$

**Figure 5:**
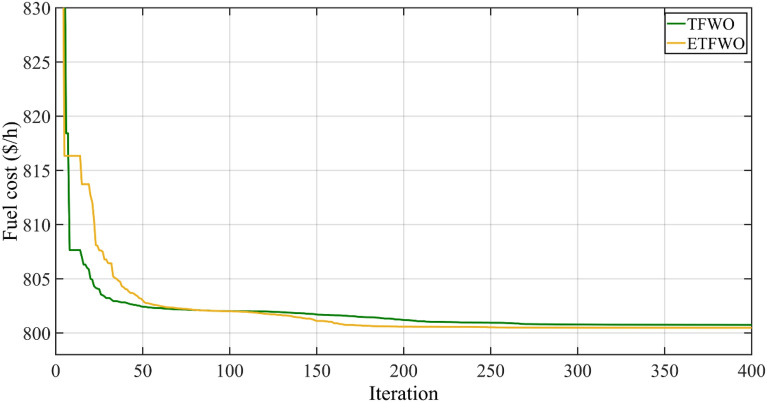
Convergence trends for Case 1.

The fuel cost using ETFWO is 800.4792 ($/h), according to simulation results shown in Table 2. In comparison to solutions from cutting-edge existing optimization approaches in Table 2, such as MSA^42^, MICA-TLA^45^, MHBMO, HFAJAYA^46^, IEP^47^, EP^43^, JAYA, GWO, DE^48^, AO^49^, MGBICA^44^, ARCBBO^50^, PSOGSA^51^, MRFO^52^, SKH^53^, TS^54^, ABC^55^, PPSOGSA^56^, SFLA-SA^57^, FA^58^, FPA^59^, MFO^60^, MPSO-SFLA^16^, and TFWO, the proposed ETFWO has meaningfully reduced the total cost. Table 3 shows that the minimum fuel cost, as determined by the ETFWO algorithm, is 800.4792 dollars per hour. Compared to the best result in the literature, the results in Table 3 show that the best fuel cost determined by the ETFWO algorithm is fairly cheap. Figure 5 displays the convergence rate for this scenario. As can be observed, the acquired data supports the capacity of the proposed ETFWO algorithm to identify precise OPF solutions in this case study.

**Table 3:** The results for Case 1: Minimizing fuel costs.

Optimizer	Fuel cost ($/h)	Emission (t/h)	Power losses (MW)	V.D. (p.u.)
MICA-TLA	801.0488	–	9.1895	–
MHBMO	801.985	–	9.49	–
AGSO	801.75	0.3703	–	–
HFAJAYA	800.4800	0.3659	9.0134	0.9047
MPSO-SFLA	801.75	–	9.54	–
IEP	802.46	–	–	–
EP	803.57	–	–	–
JAYA	800.4794	–	9.06481	0.1273
GWO	801.41	–	9.30	–
DE	802.39	–	9.466	–
MSA	800.5099	0.36645	9.0345	0.90357
AO	801.83	–	–	–
MGBICA	801.1409	0.3296	–	–
ARCBBO	800.5159	0.3663	9.0255	0.8867
PSOGSA	800.49859	–	9.0339	0.12674
MRFO	800.7680	–	9.1150	–
SKH	800.5141	0.3662	9.0282	–
FA	800.7502	0.36532	9.0219	0.9205
TS	802.29	–	–	–
ABC	800.660	0.365141	9.0328	0.9209
PPSOGSA	800.528	–	9.02665	0.91136
MFO	800.6863	0.36849	9.1492	0.75768
SFLA-SA	801.79	–	–	–
FPA	802.7983	0.35959	9.5406	0.36788
TFWO	800.7507	0.3711	9.3329	0.8940
ETFWO	800.4792	0.3662	9.0197	0.9085

#### Case 2: Minimization of piecewise quadratic fuel cost

By burning fossil fuels like coal, natural gas or petroleum, thermal generators are able to make electricity. Equation (44) provides a model for the fuel cost curve.

44 $$\begin{aligned}{J}_{2} &=\sum_{k=1}^{{n}_{f}}\sum \limits _{i=1}^{{n}_{G}}\left({a}_{i,k}+{b}_{i,k}{P}_{Gi}+{c}_{i,k}{P}_{Gi}^{2}\right)+{\lambda }_{P}\cdot {\left({P}_{G1}-{P}_{G1}^{\mathrm{lim}}\right)}^{2}+{\lambda }_{V}\sum \limits _{i=1}^{{n}_{PQ}}{\left({VL}_{i}-{VL}_{i}^{\mathrm{lim}}\right)}^{2} \\ & \quad +{\lambda }_{Q}\sum \limits _{i=1}^{{n}_{G}}{\left({Q}_{Gi}-{Q}_{Gi}^{\mathrm{lim}}\right)}^{2}+{\lambda }_{S}\sum \limits _{i=1}^{{N}_{l}}{\left({S}_{i}-{S}_{i}^{\mathrm{lim}}\right)}^{2}.\end{aligned}$$

**Figure 6:**
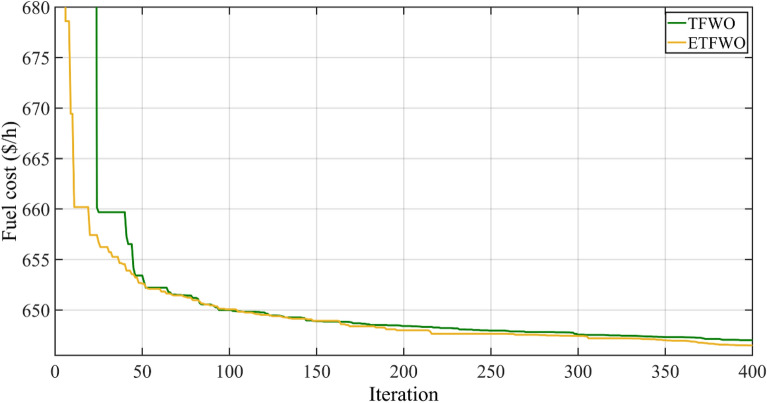
Convergence trends for Case 2.

The fuel cost in this case employing the proposed approach is 646.4860 ($/h), according to simulation results shown in Table 2. In comparison to existing optimization methods reported in recent literature shown in Table 4, such as GABC^61^, LTLBO, IEP, MFO, MICA-TLA, SSA^62^, MDE^63^, MSA, SSO, FPA, and TFWO, it is obvious that ETFWO has considerably decreased the total cost. The proposed ETFWO algorithm outperforms stochastic strategies in terms of satisfactory solutions for the OPF issues, as shown by a comparison of the results from LTLBO and the other methods listed. Table 4 shows that the minimal fuel cost is 646.4860 $/h, which is lower than the results that have been reported in the literature. The convergence trend by TFWO and ETFWO algorithms for Case 2 is shown in Fig. 6.

**Table 4:** The results for Case 2.

Optimizer	Fuel cost ($/h)	Emission (t/h)	Power losses (MW)	V.D. (p.u.)
GABC	647.03	–	6.8160	0.8010
LTLBO	647.4315	0.2835	6.9347	0.8896
IEP	649.312	–	–	–
MFO	649.2727	0.28336	7.2293	0.47024
MICA-TLA	647.1002	–	6.8945	–
SSA	646.7796	0.2836	6.5599	0.5320
MDE	647.846	–	7.095	–
MSA	646.8364	0.28352	6.8001	0.84479
SSO	663.3518	–	–	–
FPA	651.3768	0.28083	7.2355	0.31259
TFWO	647.0095	0.2837	6.8043	0.9128
ETFWO	646.4860	0.2835	6.7425	0.9235

#### Case 3: Fuel cost with VPEs

The cost function of Eq. (45) includes the valve point loading effects (VPEs).

45 $$\begin{aligned}{J}_{3} & =\sum \limits _{i=1}^{{n}_{G}}\left({a}_{i}+{b}_{i}{P}_{Gi}+{c}_{i}{P}_{Gi}^{2}+\left|{e}_{i}\mathrm{sin}\left({f}_{i}\cdot \left({P}_{Gi}^{min}-{P}_{Gi}\right)\right)\right|\right)+{\lambda }_{P}\cdot {\left({P}_{G1}-{P}_{G1}^{\mathrm{lim}}\right)}^{2}+{\lambda }_{V}\sum \limits _{i=1}^{{n}_{PQ}}{\left({VL}_{i}-{VL}_{i}^{\mathrm{lim}}\right)}^{2} \\ & \quad +{\lambda }_{Q}\sum \limits _{i=1}^{{n}_{G}}{\left({Q}_{Gi}-{Q}_{Gi}^{\mathrm{lim}}\right)}^{2}+{\lambda }_{S}\sum \limits _{i=1}^{{N}_{l}}{\left({S}_{i}-{S}_{i}^{\mathrm{lim}}\right)}^{2}.\end{aligned}$$

The fuel cost using ETFWO is 832.1625 ($/h), according to optimal results given in Table 2. For this case, ETFWO has effectively diminished the fuel cost when compared to SP-DE^64^, HFAJAYA, PSO, FA, and TFWO optimization methods in Table 5. As can be observed, the acquired data supports the capacity of the proposed ETFWO algorithm to identify precise OPF solutions in this case study. Table 5 demonstrates that the ETFWO algorithm is the best method to minimize the objective function for Case 3's OPF problem.

**Table 5:** The results for Case 3.

Optimizer	Fuel cost ($/h)	Emission (t/h)	Power losses (MW)	V.D. (p.u.)
SP-DE	832.4813	0.43651	10.6762	0.75042
HFAJAYA	832.1798	0.4378	10.6897	0.8578
PSO	832.6871	–	–	–
FA	832.5596	0.4372	10.6823	0.8539
TFWO	832.6841	0.4382	10.8556	0.8390
ETFWO	832.1625	0.4378	10.6878	0.8584

**Figure 7:**
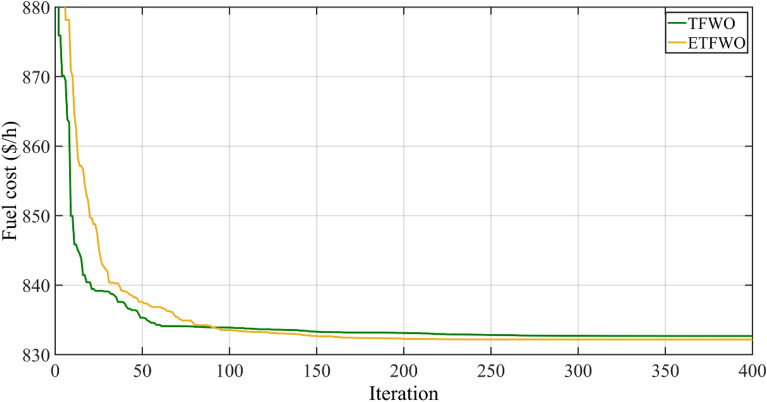
Convergence trends for Case 3.

Figure 7 depicts the convergence graph of the total cost ($/h) by the TFWO and ETFWO algorithms for Case 3.

We applied the proposed technique to Cases 4–6 in order to improve our ability to solve multi-objective OPF problems. The top ETFWO-based simulation solutions for Cases 4–6 are also summarized in Table 2.

What determines the value of these objective functions is the value of their control parameters. These control parameters are optimized and selected here by the optimization algorithm by observing all the limitations of the load dispatch problem and various constraints of the power system. As you can see in Eqs. (18), (19), and (20), the value of these functions depends on different parameters. And it is possible in the optimization that the best control parameters for one function, e.g., $VD$, give a weak value to the function ${\sum }_{i=1}^{{n}_{G}}{f}_{Ei}({P}_{Gi})$. In fact, these two objective functions are not in the same direction and have different properties. Therefore, researchers usually use multi-objective optimization, and all the objective functions are considered, making each of them give common control parameters in the algorithm's output based on their importance, which is not the best optimal state for any objective functions.

#### Case 4: fuel cost and real power loss

Using the multi-objective function provided in Eq. (46), where Eqs. (14) and (19) have been integrated to minimize both.

46 $$\begin{aligned} {J}_{4} & =\sum \limits _{i=1}^{{n}_{G}}\left({a}_{i}+{b}_{i}{P}_{Gi}+{c}_{i}{P}_{Gi}^{2}\right)+{\phi }_{p}\sum \limits _{i=1}^{{N}_{l}}\sum \limits _{\begin{array}{c}j=1\\ j\ne i\end{array}}^{{N}_{l}}\left({G}_{ij}{V}_{i}^{2}+{B}_{ij}{V}_{j}^{2}-2{V}_{i}{V}_{j}\mathrm{cos}{\delta }_{ij}\right) \\ & \quad +{\lambda }_{P}{\cdot \left({P}_{G1}-{P}_{G1}^{\mathrm{lim}}\right)}^{2}+{\lambda }_{V}\sum \limits _{i=1}^{{n}_{PQ}}{\left({VL}_{i}-{VL}_{i}^{\mathrm{lim}}\right)}^{2}+{\lambda }_{Q}\sum \limits _{i=1}^{{n}_{G}}{\left({Q}_{Gi}-{Q}_{Gi}^{\mathrm{lim}}\right)}^{2}+{\lambda }_{S}\sum \limits _{i=1}^{{N}_{l}}{\left({S}_{i}-{S}_{i}^{\mathrm{lim}}\right)}^{2},\end{aligned}$$

where, the factor ${\phi }_{p}$ is set to a value of 40 (see^42^).

**Figure 8:**
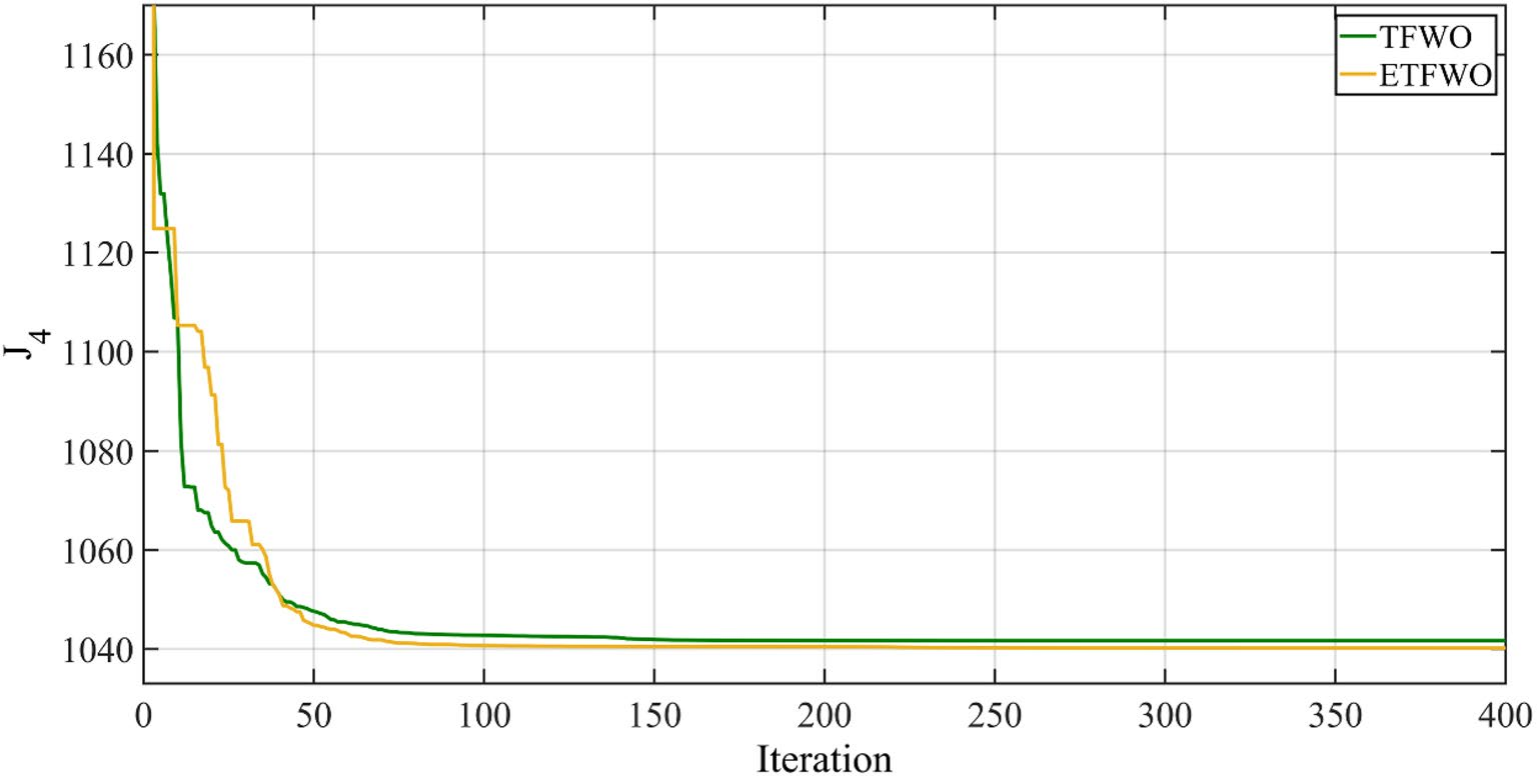
Convergence trends for Case 4.

The results represent that the fuel cost and power loss using ETFWO are 859.0080 ($/h) and 4.5295 (MW), and the recommended ETFWO provided the smallest value of the objective function, 1040.1880, which is 1.4615 less than the original TFWO's minimum value of 1041.6495. ETFWO has drastically decreased fuel cost and power loss and additionally achieved the optimum objective function compared with MJaya^36^, QOMJaya^36^, MSA, EMSA^65^, and MOALA approaches in Table 6. Figure 8 depicts the overall cost ($/h) convergence graph through the TFWO and ETFWO methodologies for Case 4.

**Table 6:** The results for Case 4.

Optimizer	Fuel cost ($/h)	Emission (t/h)	Power losses (MW)	V.D. (p.u.)	$${J}_{4}$$
MJaya	827.9124	–	5.7960	–	1059.7524
MSA	859.1915	0.2289	4.5404	0.92852	1040.8075
EMSA	859.9514	0.2278	4.6071	0.7758	1044.2354
SpDEA	837.8510	–	5.6093	0.8106	1062.223
QOMJaya	826.9651	–	5.7596	–	1402.9251
MOALA	826.4556	0.2642	5.7727	1.2560	1057.3636
TFWO	859.3015	0.2294	4.5587	0.9118	1041.6495
ETFWO	859.0080	0.2289	4.5295	0.9328	1040.1880

#### Case 5: Minimizing the fuel cost and voltage deviation

Protection of the system requires careful management of the voltage profile. Improvements to the voltage profile alleviate load bus voltage variation. Equation (47) introduces a multi-objective function for concurrently minimizing voltage deviations and fuel costs.

47 $$\begin{aligned} {J}_{5} & =\sum \limits _{i=1}^{{n}_{G}}\left({a}_{i}+{b}_{i}{P}_{Gi}+{c}_{i}{P}_{Gi}^{2}\right)+{\phi }_{v}\sum \limits _{i=1}^{{n}_{PQ}}\left|V{L}_{i}-1.0\right|+{\lambda }_{P}{\cdot \left({P}_{G1}-{P}_{G1}^{\mathrm{lim}}\right)}^{2} \\ & \quad +{\lambda }_{V}\sum \limits _{i=1}^{{n}_{PQ}}{\left({VL}_{i}-{VL}_{i}^{\mathrm{lim}}\right)}^{2}+{\lambda }_{Q}\sum \limits _{i=1}^{{n}_{G}}{\left({Q}_{Gi}-{Q}_{Gi}^{\mathrm{lim}}\right)}^{2}+{\lambda }_{S}\sum \limits _{i=1}^{{N}_{l}}{\left({S}_{i}-{S}_{i}^{\mathrm{lim}}\right)}^{2},\end{aligned}$$

where the factor ${\phi }_{v}$ is set to a value of 100 (see^43^).

**Figure 9:**
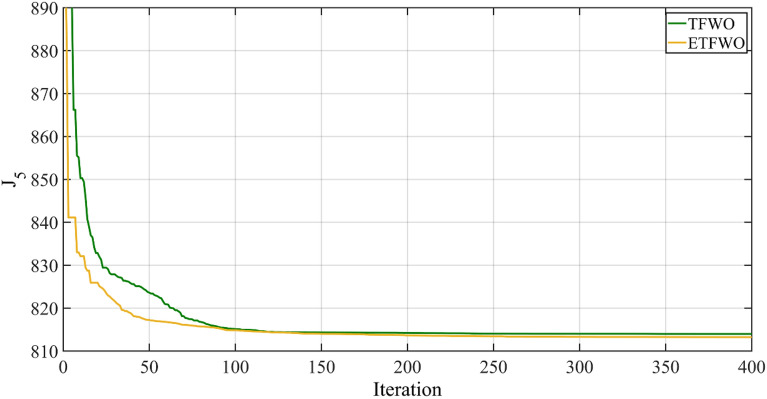
Convergence trends for Case 5.

Using the proposed method, we obtained results that reveal that the fuel cost and voltage discrepancies are 803.7868 dollars per hour and 0.0945 per unit, respectively. The multi-objective fitness function that ETFWO discovered to have a minimum value of 813.2368 is the smallest and best one available compared to SpDEA^66^, SSO, PSO^67^, PSO-SSO^67^, MPSO^68^, BB-MOPSO^69^, MOMICA^70^, MNSGA-II^71^, EMSA, DA-APSO^72^, TFWO, and TFWO approaches, which is shown in Table 7. Figure 9 depicts the convergence graph of the overall cost ($/h) through the TFWO and ETFWO methodologies for Case 5.

**Table 7:** The results for Case 5.

Optimizer	Fuel cost ($/h)	Emission (t/h)	Power losses (MW)	V.D. (p.u.)	$${J}_{5}$$
PSO	804.477	0.368	10.129	0.126	817.0770
BB-MOPSO	804.9639	–	–	0.1021	815.1739
MOMICA	804.9611	0.3552	9.8212	0.0952	814.4811
MNSGA-II	805.0076	–	–	0.0989	814.8976
SSO	803.73	0.365	9.841	0.1044	814.1700
EMSA	803.4286	0.3643	9.7894	0.1073	814.1586
PSO-SSO	803.9899	0.367	9.961	0.0940	813.3899
DA-APSO	802.63	–	–	0.1164	814.2700
TFWO	803.416	0.365	9.795	0.101	813.5160
SpDEA	803.0290	–	9.0949	0.2799	831.0190
MPSO	803.9787	0.3636	9.9242	0.1202	815.9987
TFWO	804.1048	0.3639	10.1895	0.0988	813.9848
ETFWO	803.7868	0.3638	9.8694	0.0945	813.2368

#### Case 6: Minimizing the fuel cost, emissions, voltage deviation and losses

This problem, provided by Eq. (48), integrates instances 1, 5, and 6 to simultaneously reduce fuel expenditure, voltage variation, emission, and power loss.

48 $$\begin{aligned}{J}_{6} &=\sum \limits _{i=1}^{{n}_{G}}\left({a}_{i}+{b}_{i}{P}_{Gi}+{c}_{i}{P}_{Gi}^{2}\right)+{\phi }_{p}\sum \limits _{i=1}^{{N}_{l}}\sum \limits _{\begin{array}{c}j=1\\ j\ne i\end{array}}^{{N}_{l}}\left({G}_{ij}{V}_{i}^{2}+{B}_{ij}{V}_{j}^{2}-2{V}_{i}{V}_{j}\mathrm{cos}{\delta }_{ij}\right) \\ & \quad +{\phi }_{v}\sum \limits _{i=1}^{{n}_{PQ}}\left|V{L}_{i}-1.0\right|+{\phi }_{e}\sum \limits _{i=1}^{{n}_{G}}\left({\alpha }_{i}+{\beta }_{i}{P}_{Gi}+{\gamma }_{i}{P}_{Gi}^{2}+{\xi }_{i}\mathrm{exp}({\theta }_{i}{P}_{Gi})\right)+{\lambda }_{P}{\left({P}_{G1}-{P}_{G1}^{\mathrm{lim}}\right)}^{2} \\ & \quad +{\lambda }_{V}\sum \limits _{i=1}^{{n}_{PQ}}{\left({VL}_{i}-{VL}_{i}^{\mathrm{lim}}\right)}^{2} +{\lambda }_{Q}\sum \limits _{i=1}^{{n}_{G}}{\left({Q}_{Gi}-{Q}_{Gi}^{\mathrm{lim}}\right)}^{2}+{\lambda }_{S}\sum \limits _{i=1}^{{N}_{l}}{\left({S}_{i}-{S}_{i}^{\mathrm{lim}}\right)}^{2}.\end{aligned}$$

The weight variables are used to manage the problem’s several objectives as in^42^ with ${\phi }_{v}=21,$ ${\phi }_{p}=22,$ and ${\phi }_{e}=19$.

**Figure 10:**
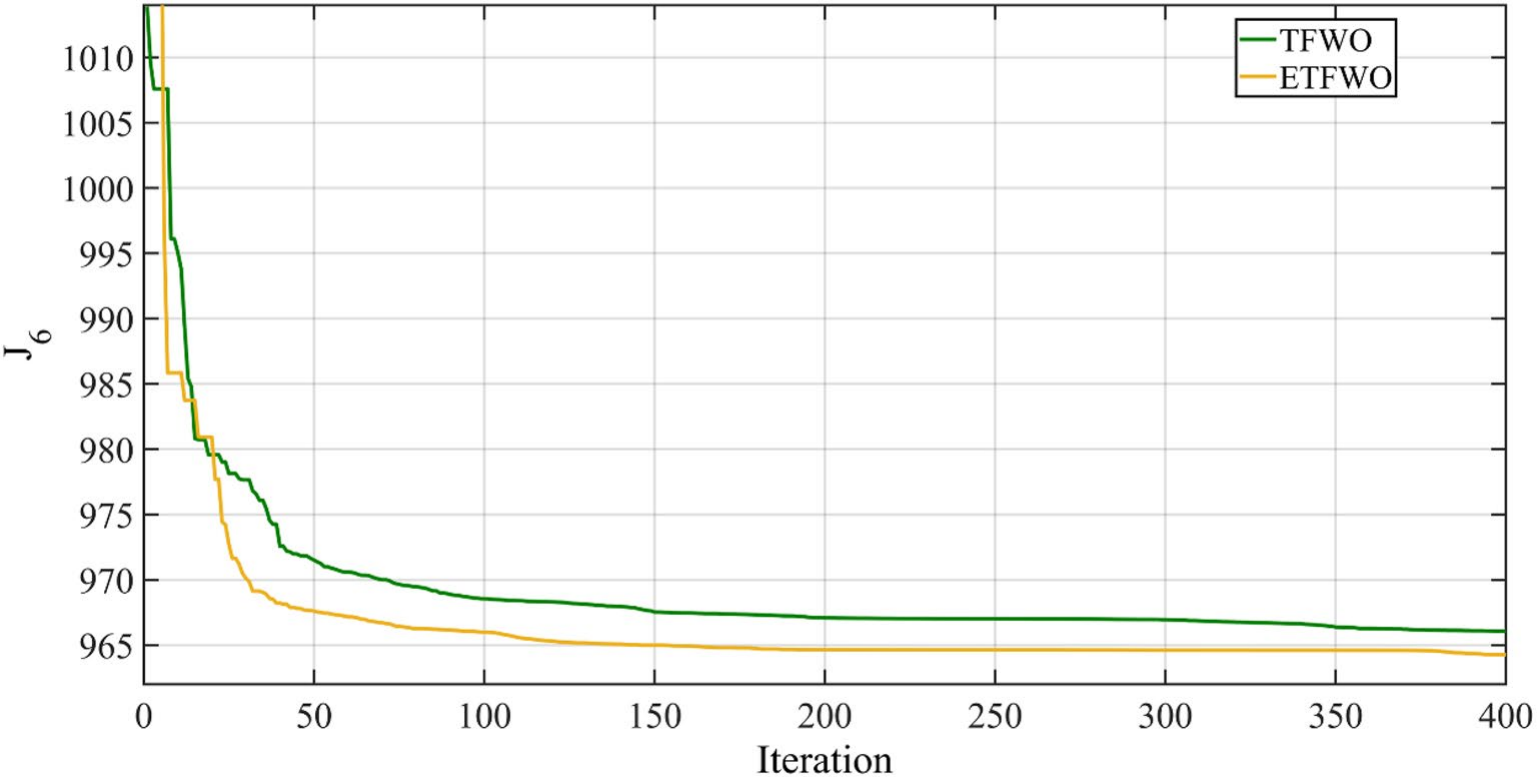
Convergence trends for Case 6.

The simulation findings depicted in Table 8 demonstrate that ETFWO has greatly optimized all the objects compared to MNSGA-II, MOALA, J-PPS1^73^, J-PPS2^73^, J-PPS3^73^ , SSO, MSA, BB-MOPSO, PSO, MODA^74^, I-NSGA-III^75^, MFO, and TFWO approaches. This table demonstrates that, in this example, the proposed ETFWO optimizer outperformed the other optimization techniques. Table 8 demonstrates that the suggested ETFWO's objective function ${{\varvec{J}}}_{6}$, which is 964.2683, is the lowest of all the approaches. Figure 10 illustrates the total cost convergence graph for Case 6 for both TFWO and ETFWO.

**Table 8:** The results for Case 6.

Algorithm	Fuel cost ($/h)	Emission (t/h)	Power losses (MW)	V.D. (p.u.)	$${J}_{6}$$
J-PPS2	830.8672	0.2357	5.6175	0.2948	965.1201
MOALA	826.2676	0.2730	7.2073	0.7160	1005.0512
J-PPS1	830.9938	0.2355	5.6120	0.2990	965.2159
SSO	829.978	0.25	5.426	0.516	964.9360
MSA	830.639	0.25258	5.6219	0.29385	965.2907
BB-MOPSO	833.0345	0.2479	5.6504	0.3945	970.3379
J-PPS3	830.3088	0.2363	5.6377	0.2949	965.0228
PSO	828.2904	0.261	5.644	0.55	968.9674
MFO	830.9135	0.25231	5.5971	0.33164	965.8080
MNSGA-II	834.5616	0.2527	5.6606	0.4308	972.9429
MODA	828.49	0.265	5.912	0.585	975.8740
I-NSGA-III	881.9395	0.2209	4.7449	0.1754	994.2078
TFWO	831.1836	0.2547	5.6221	0.3028	966.0679
ETFWO	830.2325	0.2530	5.5913	0.2962	964.2683

In Cases 1–6, the presented approach did a better job of exploring than newly reported methods that seem to be trapped at a local solution. Overall, the performance of TFWO and ETFWO is quite competitive and consistently outperforms most algorithms.

### The solution of OPF involving WT and PV producers in the IEEE 30 bus test system

#### Case 7: Cost-cutting through the use of stochastic wind and solar power

In this scenario, the goal is to employ the ETFWO to get the fuel, wind, and PV costs from Eq. (49) to be as low as possible for a system that uses renewable energy sources like solar and wind.

49 $$\begin{aligned}{J}_{7} &=\sum \limits _{i=1}^{{n}_{G}}\left({a}_{i}+{b}_{i}{P}_{Gi}+{c}_{i}{P}_{Gi}^{2}\right)+\sum \limits _{i=1}^{{n}_{v}}\left({{C}_{d,pv,i}+C}_{ue,pv,i}+{C}_{oe,pv,i}\right)+\sum \limits _{i=1}^{{n}_{w}}\left({C}_{d,w,i}+{C}_{ue,w,i}+{C}_{oe,w,i}\right) \\ & \quad +{\phi }_{v}\sum \limits _{i=1}^{{n}_{PQ}}\left|V{L}_{i}-1.0\right|+{\lambda }_{P}{\cdot \left({P}_{G1}-{P}_{G1}^{\mathrm{lim}}\right)}^{2} \\ & \quad +{\lambda }_{V}\sum \limits _{i=1}^{{n}_{PQ}}{\left({VL}_{i}-{VL}_{i}^{\mathrm{lim}}\right)}^{2}+{\lambda }_{Q}\sum \limits _{i=1}^{{n}_{G}}{\left({Q}_{Gi}-{Q}_{Gi}^{\mathrm{lim}}\right)}^{2}+{\lambda }_{S}\sum \limits _{i=1}^{{N}_{l}}{\left({S}_{i}-{S}_{i}^{\mathrm{lim}}\right)}^{2}.\end{aligned}$$

The cost coefficients for this type of OPF have been chosen the same as in Case 1 with the PDF parameters in^11^.

The best optimal solutions obtained from 30 independent runs by the TFWO and ETFWO algorithms in this article are given in Table 9. It is worth mentioning that ${P}_{ws1}$ shows the wind generator planned power ${W}_{G1}$, and so on.

**Table 9:** The optimal values of the variables were achieved for Case 7.

Variables	TFWO	ETFWO
$${P}_{G1}$$ (MW)	134.9079	134.9079
$${P}_{G2}$$ (MW)	29.17	28.2463
$${P}_{ws1}$$ (MW)	44.106	43.6341
$${P}_{G3}$$ (MW)	10	10
$${P}_{ws2}$$ (MW)	37.2285	36.8123
$${P}_{SS}$$ (MW)	33.755	35.6711
$${V}_{G1}$$ (p.u.)	1.0718	1.0736
$${V}_{G2}$$ (p.u.)	1.0569	0.95
$${V}_{G5}$$ (p.u.)	1.035	1.0379
$${V}_{G8}$$ (p.u.)	1.0595	1.1
$${V}_{G11}$$(p.u.)	1.0981	1.1
$${V}_{G13}$$ (p.u.)	1.0488	1.0635
$${Q}_{G1}$$ (MVAR)	$$-2.3090$$	16.6759
$${Q}_{G2}$$ (MVAR)	11.8332	$$-20$$
$${Q}_{ws1}$$ (MVAR)	22.417	30.1178
$${Q}_{G3}$$ (MVAR)	40	40
$${Q}_{ws1}$$(MVAR)	30	30
$${Q}_{ss}$$ (MVAR)	15.0612	20.6142
Fuel valve cost ($/h)	442.8016	439.7259
Wind gener. cost ($/h)	248.4560	245.3895
Solar gener. cost ($/h)	91.2925	96.8096
Total cost ($/h)	782.5501	781.9250
Emission (t/h)	1.7619	1.7621
Power losses (MW)	5.7674	5.8717
*V.D.* (p.u.)	0.45395	0.49559

**Figure 11:**
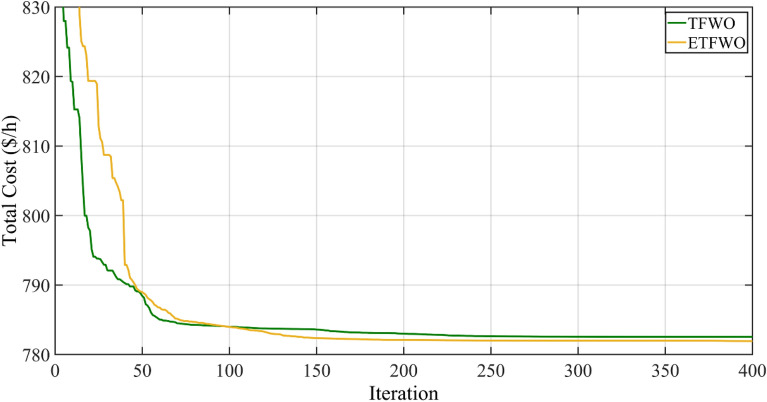
Convergence trends for Case 7.

As it is clear from this table, the proposed algorithm of ETFWO was able to achieve optimal solutions with more quality than the TFWO algorithm. The best total cost was achieved by the proposed ETFWO, which came in at 781.9250 ($/h), which is 0.6251 less than the original TFWO's minimum total cost of 782.5501 ($/h). Figure 11 depicts the total cost convergence graph for Case 7.

#### Case 8: OPF with carbon tax

This case study aims to lower total generation costs by placing a carbon price on traditional thermal power companies' emissions. It is well known that conventional energy sources create hazardous gases when used to generate electricity. In recent years, a great deal of pressure has been placed on the energy industry, in general, to cut carbon emissions by numerous nations. The reason for this is global warming.

Carbon tax (${C}_{\mathrm{tax}}$) is imposed on each generator of greenhouse gas emissions to encourage investment in solar and wind. The value of emissions^11^ is calculated by:

50 $${C}_{E}={C}_{\mathrm{tax}}\cdot E,$$

51 $${J}_{8}={J}_{7}+{C}_{tax}E,$$

where $${C}_{\mathrm{tax}}$$ is expected to cost $20 per tonne per hour (t/h).

**Figure 12:**
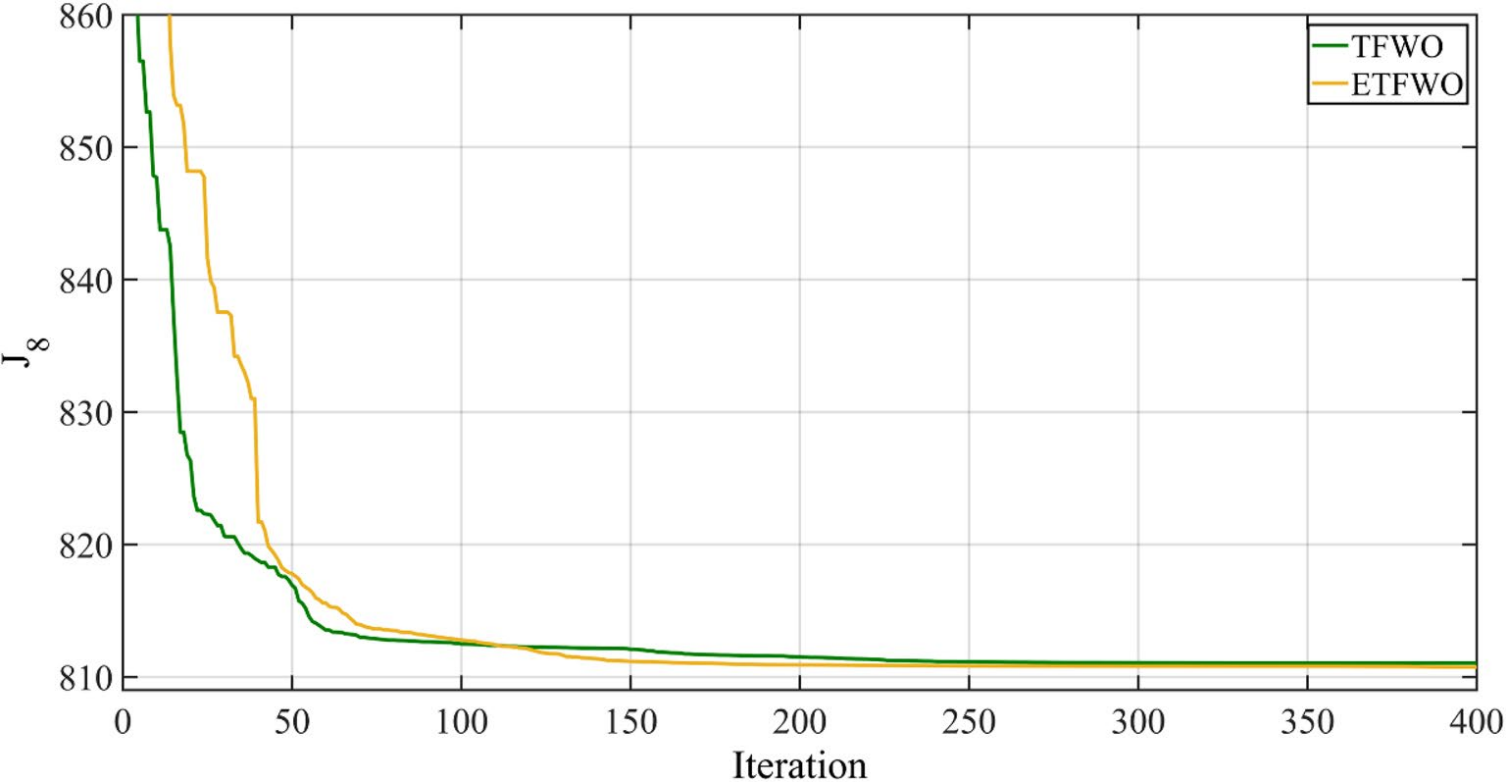
Convergence trends for Case 8.

Table 10 displays the optimal generation schedule, reactive generator power, total generation cost, and additional calculated factors. In Case 8, where a carbon tax has been imposed, wind and solar energy penetration are greater than in Case 7, in which no carbon tax is regarded. The amount of growth in the optimal schedule for producing renewable energy is contingent on the number of emissions and the speed with which a carbon price is implemented. As it is clear from this table, the best objective function, 810.7341, was produced by the proposed ETFWO, which is 0.3177 less than the 811.0518 minimum value of the objective function obtained by the original TFWO. Figure 12 also displays the TFWO and ETFWO convergence rate for Case 8 of this study.

**Table 10:** The optimal values of the variables were achieved for Case 8.

Variables	TFWO	ETFWO
*P*_*G1*_ (MW)	123.85266	123.03317
*P*_*G2*_ (MW)	33.9622	31.763
*P*_*ws1*_ (MW)	46.4927	45.3487
*P*_*G3*_ (MW)	10	10
*P*_*ws2*_ (MW)	39.1298	38.2016
*P*_*ss*_ (MW)	35.2403	40.3302
*V*_*G1*_ (p.u.)	1.0709	1.0702
*V*_*G2*_ (p.u.)	1.0574	1.0567
*V*_*G5*_ (p.u.)	1.0363	1.0355
*V*_*G8*_ (p.u.)	1.0404	1.0403
*V*_*G11*_ (p.u.)	1.0983	1.0985
*V*_*G13*_ (p.u.)	1.0552	1.0570
*Q*_*G1*_ (MVAR)	− 2.59156	− 2.79228
*Q*_*G2*_ (MVAR)	12.4069	12.1838
*Q*_*ws1*_ (MVAR)	22.9491	22.9875
*Q*_*G3*_ (MVAR)	35.3754	35.1216
*Q*_*ws2*_ (MVAR)	30	30
*Q*_*ss*_ (MVAR)	17.518	18.1894
Fuelvlvcost ($/h)	433.4367	423.9783
Wind gen cost ($/h)	263.5752	256.2065
Solar gen cost ($/h)	95.8757	113.2142
Total Cost ($/h)	792.8876	793.3989
Emission (t/h)	0.90821	0.86676
*J*_8_	811.0518	810.7341
Power losses (MW)	5.2777	5.2766
V.D. (p.u.)	0.46785	0.47126
Carbon tax ($/h)	18.1642	17.3352

When addressing the OPF problem while considering the integration of stochastic wind and solar power, the proposed ETFWO demonstrates excellent resilience and superior efficiency compared to the original TFWO and other recent techniques based on the analysis of the optimal results in Cases 7 and 8.

### ETFWO for different OPF problems in the IEEE 118 test system

The effectiveness of the proposed algorithm was assessed utilizing an extensive power system. Its outcomes were compared to those of the other approved algorithms to ensure the suggested strategy's viability.

#### Case 9: Classical OPF

Comparing the IEEE 30-bus system, the number of control variables rises from 24 to 130. Adding more generators makes the OPF issue more challenging. The system needs 4242 MW of active power and 1439 MVAR of reactive power, respectively. The aim function in instances 1 and 9 is to minimize the fuel cost in order to be comparable to other cutting-edge heuristic algorithms presented in the literature. The top results obtained with the suggested ETFWO method are listed in Table 11. Table 11 compares this finding to the outcomes of other algorithms being studied and a few other strategies that have been published in the literature, including MSA^52^, FPA^52^, MFO^52^, PSOGSA^52^, IABC^76^, MCSA^77^, MRao-2 and Rao algorithms^78^, SSO^37^, FHSA^64^, ICBO^3^, GWO^23^, EWOA^79^, and CS-GWO^80^.

**Table 11:** Optimal results for Case 9.

Optimizer	Min	Mean	Max	Std	Time (s)
ETFWO	129,542.8215	129,550.8843	129,561.7019	7.13	723.76
TFWO	129,695.1429	129,768.5270	–129,849.5436	167.52	750.37
MCSA^79^	129,873.6	–	–	–	–
IABC^76^	129,862.0	129,895.0	–	40.8	4157.8
PSOGSA^52^	129,733.6	–	–	–	–
MFO^52^	129,708.1	–	–	–	–
ICBO^3^	135,121.6	–	–	–	–
FHSA^78^	132,138.3	132,138.3	132,138.3	0.0	–
SSO^78^	132,080.4	–	–	–	–
FPA^52^	129,688.7	–	–	–	–
MSA^52^	129,640.7	–	–	–	–
CS-GWO^80^	129,544.0	10.7	4252.5		
Rao-1^79^	131,817.9	–	–	–	808.0
EWOA^80^	140,175.8	–	–	–	–
Rao-3^78^	131,793.1	–	–	–	806.7
Rao-2^78^	131,490.7	–	–	–	804.6
GWO^23^	139,948.1	142,989.3	145,484.6	797.8	1766.2
MRao-2^78^	131,457.8	–	–	–	1160.3

Table 11 shows that the ETFWO performs better than several optimization methods for solving large-scale OPF. Working with such a massive system, the CS-GWO delivers remarkable results.

## Discussions

All cases that were looked into by both TFWO and ETFWO were put through a comparison study with other well-known algorithms, including WSO (war strategy optimization)^81^, HHO (Harris Hawks optimizer)^82^, and FDA (flow direction algorithm)^83^ in this section. Reliability evaluation is a non-empirical way to confirm something and is essential for studying complex things. For Cases 1 and 8, a sensitivity analysis was performed to see how stable the metaheuristics under consideration were. Table 12 shows the results of this analysis according to the best value of the solution (Min), the average value of the solutions (Mean), the worst solution obtained (Max), the standard value of the standard deviation (Std.), and the median value of the time of 30 data runs. This table demonstrates that the proposed ETFWO algorithm is more stable and reliable than the TFWO, WSO, HHO, and FDA algorithms. The ETFWO algorithm surpassed the examined algorithms in terms of Min, Mean, Max, and Std. values for all the objective functions of OPF, proving its superior efficiency. Furthermore, the proposed ETFWO algorithm came out on top in every OPF task, demonstrating its better performance than the alternatives. The TFWO and FDA algorithms showed strong efficacy, placing second and third for the majority of cases, respectively.

**Table 12:** Optimal results to represent the performance of algorithms.

Method	Min	Mean	Max	Std	Time (s)
	Case 1				
ETFWO	800.4792	800.5683	800.7065	0.18	24
TFWO	800.7507	800.9715	801.2241	0.35	21
WSO	801.6528	802.2390	803.2471	1.36	26
HHO	801.3894	801.9856	802.7006	0.97	35
FDA	800.9037	801.5543	802.2595	1.04	28

Method	Min	Mean	Max	Std	Time (s)
	Case 2				
ETFWO	646.4860	646.6107	646.7329	0.15	24
TFWO	647.0095	647.3481	647.6720	0.28	26
WSO	647.4814	647.8963	648.3419	0.87	25
HHO	647.4526	647.7349	648.1202	0.91	38
FDA	647.2133	647.5618	647.9018	0.64	27

Method	Min	Mean	Max	Std	Time (s)
	Case 3				
ETFWO	832.1625	832.2832	832.4109	0.14	28
TFWO	832.6841	832.8940	833.1738	0.39	30
WSO	833.2117	833.4696	834.0074	0.76	28
HHO	833.2451	833.4529	834.2149	0.92	34
FDA	832.8945	833.1854	833.5471	0.58	30

Method	Min	Mean	Max	Std	Time (s)
	Case 4				
ETFWO	1040.1880	1040.3716	1040.5073	0.19	27
TFWO	1041.6495	1041.9030	1042.3118	0.27	23
WSO	1041.5812	1042.2794	1042.6340	0.84	26
HHO	1041.4799	1041.8714	1042.5843	0.96	35
FDA	1041.8341	1042.1869	1042.7828	1.24	28

Method	Min	Mean	Max	Std	Time (s)
	Case 5				
ETFWO	813.2368	813.4013	813.5238	0.21	26
TFWO	813.9848	814.2247	814.6811	0.47	25
WSO	814.1784	814.4637	814.7624	0.75	30
HHO	814.3572	814.6482	815.4237	1.03	38
FDA	814.0852	814.3816	814.9020	0.81	25

Method	Min	Mean	Max	Std	Time (s)
	Case 6				
ETFWO	964.2683	964.4062	964.5570	0.17	28
TFWO	966.0679	966.3681	966.6321	0.32	30
WSO	965.4136	966.4525	967.1790	0.64	33
HHO	966.2418	967.5014	968.2416	0.75	36
FDA	965.3849	965.9230	967.1596	1.45	30

Method	Min	Mean	Max	Std	Time (s)
	Case 7				
ETFWO	781.9250	782.2788	782.4619	0.12	27
TFWO	782.5501	782.9105	783.2687	0.30	29
WSO	782.7862	782.9746	783.3415	0.57	32
HHO	783.2568	783.8004	784.1969	0.95	40
FDA	782.8026	783.1546	783.6644	0.63	32

Method	Min	Mean	Max	Std	Time (s)
	Case 8				
ETFWO	810.7341	810.8554	810.9732	0.15	24
TFWO	811.0518	811.2975	811.5884	0.28	24
WSO	811.8724	812.3361	812.9063	0.72	28
HHO	811.6146	812.1595	812.8200	0.69	34
FDA	811.5239	811.8249	813.0819	1.23	30

## Conclusions

ETFWO is a new enhanced turbulent flow of water-based optimization (TFWO) method that has been proposed, developed, and used effectively to rectify eight various testing cases of OPF problems in the IEEE 30-bus system with a mix of photovoltaic units and energy of wind. The findings demonstrate that the current metaheuristic works well for large-scale applications because it quickly converges and doesn't get stuck at local minima very often. Solutions analysis and a comparative study with recently published OPF methods showed that ETFWO is a valid, effective, and robust method for calculating a set of steady optimal solutions for a hybrid electrical network under real-world conditions. This is a very important part of running modern power systems, which use a growing number of different types of energy. The proposed metaheuristic did better than current well-known optimizers, which proves that it is better and has the potential to yield reliable and precise remedies for multi-objective optimization. In fact, ETFWO could be used as a tool to respond to many particular questions about large, complex systems in general, which would lead to more research. The outcomes demonstrated the applicability and potential of the proposed algorithms for resolving various MOOPF problems. Simulation results validated the ability of the proposed algorithms to produce accurate and well-distributed results for all multi-objective optimization problems considered. Based on the findings of this study, the proposed ETFWO optimization tools are efficient, trustworthy, and quick at solving various MOOPF problems. Environmentally speaking, the proposed optimization technique results in the lowest emission levels. In particular, the proposed ETFWO algorithm is an excellent candidate for solving MOOPF problems in real-world power systems due to its high-quality solutions and excellent convergence properties. In addition, the comparative analysis with the proposed algorithms and published OPF solution methods validates the superiority of the proposed paradigm and its ability to locate valid and accurate solutions, particularly for multi-objective optimization problems.

The ETFWO optimization technique can be used to find a solution to the OPF problem for the future, considering the power system's unpredictability and its components (such as load demand and alternative renewable energy sources). To improve the quality of solutions in the optimization domain, researchers have proposed creating a new hybrid version of the ETFWO algorithm by combining it with other optimization methods, such as PSO and DE approaches. However, the anticipated research activity will extend into the future to examine the ranking of contingencies and the placement of scattered generations. In conclusion, AI algorithms present a formidable new tool for solving complex engineering optimization and MOOPF challenges.

## Data Availability

All data generated or analyzed during this study are included directly in the text of this submitted manuscript. There are no additional external files with datasets.
